# Simple Does Not Mean Trivial: Behavior of Phosphatidic Acid in Lipid Mono- and Bilayers

**DOI:** 10.3390/ijms222111523

**Published:** 2021-10-26

**Authors:** Dominik Drabik, Aleksander Czogalla

**Affiliations:** Laboratory of Cytobiochemistry, Faculty of Biotechnology, University of Wroclaw, F. Joliot-Curie 14a, 50-383 Wroclaw, Poland

**Keywords:** phosphatidic acid, molecular dynamics, flicker noise spectroscopy, Langmuir monolayers, bending rigidity, area compressibility, free energy of mixing

## Abstract

Phosphatidic acid (PA) is one of the simplest membrane phospholipids, yet it plays a crucial role in various biologically relevant processes that take place in cells. Since PA generation may be triggered by a variety of factors, very often of antagonistic character, the specific nature of physiological responses driven by PA is not clear. In order to shed more light on these issues, we carried out a systematic characterization of membranes containing one of the three biologically significant PA molecular species. The effect of these molecules on the properties of membranes composed of phosphatidylcholine and/or cholesterol was assessed in a multidisciplinary approach, including molecular dynamic simulations, flicker noise spectroscopy, and Langmuir monolayer isotherms. The first enables the determination of various macroscopic and microscopic parameters such as lateral diffusion, membrane thickness, and defect analysis. The obtained data revealed a strong interaction between unsaturated PA species and phosphatidylcholine. On the other hand, the behavior of saturated PA was greatly influenced by cholesterol. Additionally, a strong effect on mechanical properties was observed in the case of three-component systems, which could not be explained by the simple extrapolation of parameters of the corresponding two-component systems. Our data show that various PA species are not equivalent in terms of their influence on lipid mono- and bilayers and that membrane composition/properties, particularly those related to the presence of cholesterol, may strongly modulate PA behavior.

## 1. Introduction

Phosphatidic acid (PA) is one of the simplest phospholipids. Yet it is characterized by unique biophysical (physicochemical) properties: a small headgroup; negative charge and a phosphomonoester group. Its cellular content is rather small, as it is estimated to constitute about 1–4 mol% of all lipids. However, it plays a crucial role in lipid metabolism, as it is used in the synthesis of all other types of glycerophospholipids and acts as a modulator of membrane shape [1,2]. It is an important second messenger, capable of binding and/or activating a broad range of effector proteins thus influencing their functionality [3]. Its interaction network includes more than 50 different proteins such as kinases, phosphatases, and nucleotide-binding proteins, to name a few [4]. Additionally, PA was reported to increase the binding of neurotransmitters and anesthetics to lipid membranes [5]. Nevertheless, despite numerous studies, it still remains poorly understood what determines the specificity of PA recognition by proteins [6,7]. It is worth underlining that recognition of lipidic components of membranes should not be interpreted as simple receptor-ligand binding events but also take into account physicochemical properties of the membrane. For example, it was suggested that PA–protein binding is dependent on the membrane curvature stress [6,8], especially as PA, due to its shape, is believed to form negative membrane curvature. Furthermore, hydrogen bond formation was observed as a phenomenon that takes place during interaction with membranes with PC:PE mixed lipids, especially when proteins were present [1,8,9]. Thus, there is a need to study lipid–protein interactions in the context of detailed properties of the lipid bilayer.

It is not surprising that changes in membrane composition may have a significant influence on its properties and behavior [10,11]. This can result in changes for instance in membrane curvature, mechanical bending, compressibility, thickness, lateral diffusion, and/or local water permeability. Changes in lipid composition may also contribute to the formation of membrane defects and changes in lipid headgroup conformation and/or presentation at the membrane interface, all of which were reported, along with membrane curvature, to modulate protein recruitment [11,12,13,14]. The local concentration of PA may significantly exceed the average concentration due to transient activation of a number of PA-producing enzymes [1]. Significantly, PA as a cone-shaped lipid also has the ability to induce membrane defect formation [15]. Such local change of membrane properties could result in a locally different state of the membrane that is recognizable by certain proteins. To this end, it is crucial to determine which properties of the membrane are influenced by the presence of PA molecules. The determination of such properties could provide a link between biophysical studies of membrane properties and the molecular mechanisms underlying protein–membrane interactions, which still await detailed description. From this perspective a protein is interacting with the local membrane area—an object with given physicochemical properties—rather than an individual lipid molecule [11]. However, the proposed classification of lipids based only on lipid head is not sufficient to discuss various biological phenomena. Kassas et al. [16] showed that acyl chain length and saturation could also be important factors for selectivity when investigating phosphatidic acid sensors.

In this study, we focused on filling that void and characterized the membranes with phosphatidic acids such as POPA (16:0–18:1), DPPA (16:0–16:0), and SAPA (18:0–20:4). The choice of these three PA species was guided by their biological relevance. Specifically, they are selected based on predominant metabolic pathways found in mammalian cells. The product of the diacylglycerol kinase pathway is mostly SAPA, while lysophosphatidic acid acetyltransferase provides short length DPPA and phospholipase D focuses on transforming PC lipids (of which POPC is the most common) to PA lipids. Additionally, they differ in their chemical structure, namely acyl chain lengths and level of saturation. In consequence, they differ significantly in transition temperature (T_m_). For DPPA, the room temperature is far below its T_m_, which suggests that in our experimental conditions it is in the gel phase; POPA is close to its T_m_ and SAPA is far above its T_m_, and hence stays in the fluid phase. Our characterization includes studies of topological, mechanical, and dynamic aspects of monolayers and bilayers containing these lipids. Specifically, we focused on the effect of various PA lipid molecules on membranes with POPC and/or cholesterol. POPC was selected as the most common PC lipid in the membranes of mammalian cells [17]. Cholesterol, on the other hand, is the most biologically relevant mechanical modulator of membranes of animal cells [18]. It is believed to maintain the integrity and fluidity of cell membranes as well as serving as a precursor for the synthesis of vital substances. At physiological concentrations (35 mol% in the plasma membrane) it can induce different phases in lipid membranes and influence the conformation and function of proteins in the membrane [11]. Remarkably, the effect of cholesterol is very often neglected in studies on the influence of phosphatidic acid on mechanical features of lipid bilayers and on the specificity of protein–membrane interactions.

In this work, we combined molecular dynamics (MD) simulations with experimental approaches, such as flicker noise spectroscopy for the description of mechanical behavior of the membrane and Langmuir monolayer isotherms for detailed characterization of membrane phenomena related to PA. The theoretical approach allowed us to expand the characterization with a more detailed analysis of various microscopic parameters and focus on the interaction with POPC and/or cholesterol membrane components. Our research shows that both POPA and SAPA interact strongly with POPC molecules, while DPPA prefers cholesterol. This was reflected by various parameters such as membrane thickness and area per lipid as well as mixing character of the monolayer systems. Additionally, we systematically characterized three-components membranes, which are not commonly investigated although more relevant to physiological conditions. It allowed us to discover an unexpected effect on the mechanical properties of membranes. We believe that our work could shed more light not only on the specific details of lipid–lipid interactions but also on molecular mechanisms that govern protein recruitment to membranes, vesicle biogenesis and turnover, to name a few.

## 2. Results and Discussion

### 2.1. Molecular Dynamics Characterization

**Figure 1:**
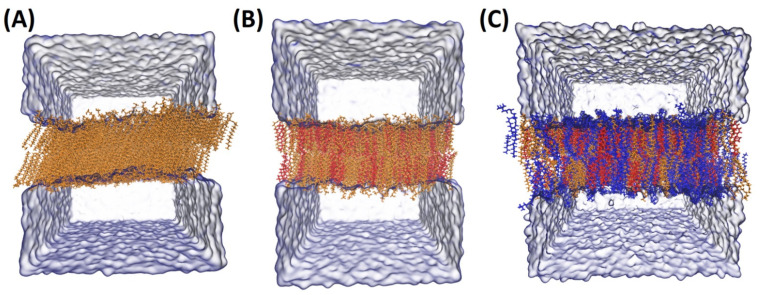
Snapshots of membrane MD systems. In panels (**A**) DPPA, (**B**) Chol/DPPA 1:1 (**C**) POPC/Chol/SAPA 5:3:2 (molar ratios) are shown. Molecules are colored according to the following rule: orange is PA component, red is cholesterol, and blue is POPC lipid molecules. As periodic boundary conditions were applied, the water molecules are organized with respect to periodic repetition of the system (gray/light blue).

All lipid membranes systems were subjected to detailed characterization of their topological and mechanical properties. Parameters of bulk membranes are summarized in Table 1. More detailed information regarding structures of lipids and a table detailing individual lipid species and leaflets is presented in supporting information (Figures S1 and S2, and Table S1). Selected snapshots of simulated systems are presented in Figure 1. Defect analysis was performed, as it was suggested that the presence of defects in the headgroup region (or increased acyl chain accessibility) of lipid bilayers exert an important effect on interactions of peripheral membranes with PA-containing membranes [19,20]. Simple measurement of headgroup surface integrity is the fraction of the surface map that shows the exposed acyl chain region F_exp_. However, as observed by Boyd et al. [19], the total exposure of chains does not take into account the geometry of the gaps in the headgroup region, which is relevant to protein binding. To this end, the fraction of exposed acyl chains in the function of the radius of the probe sphere is analyzed. To enhance the clarity of data description, three aspects were considered: the effect of PA on POPC, cholesterol, or POPC/cholesterol membrane systems. In this subsection, only parameters exclusive to MD will be discussed. Both area per lipid (APL) and bending rigidity will be discussed in subsequent subsections with the respective experimental data.

**Figure 2:**
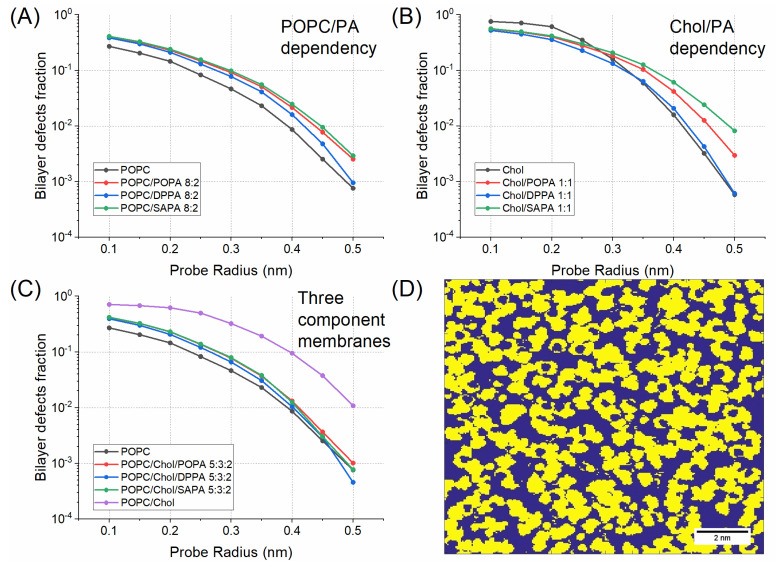
Fraction of exposed acyl chains in function of probe radius for (**A**) POPC/PA, (**B**) Chol/PA, and (**C**) POPC/Chol/PA membrane systems. (**D**) A top-down probed surface grid of a frame in the POPC/SAPA bilayer simulation. On the grid, yellow represents the headgroup region, while blue represents the acyl chains region. The surface is probed with 0.2 nm radius size.

The effect of PA lipids on the POPC membrane is, in general, consistent for all of the investigated PA species. When PA molecules were present in the membrane, in all cases membrane thickness (MT) was significantly increased. Similarly, the tilt and lateral diffusion coefficients of the system were also increased. The latter parameter, in particular, directly correlated with membrane curvature, which was described as a potential factor for peptide–lipid interactions [8,9]. Interestingly, interdigitation increased for POPA and SAPA but decreased for DPPA. This could be, however, due to DPPA being in the gel phase, especially that membranes in the gel phase are known to become thicker. Furthermore, the area compressibility coefficient (K_A_) increased for both POPA and DPPA but greatly decreased for SAPA, which indicates that SAPA has a weakening effect on membrane resilience. Defect analysis shows that each PA used in our study increased the fraction of exposed acyl chains (see Figure 2A). This effect was stronger in the case of POPA and SAPA membranes. In general, in our approach the greater the radius of the sphere probe, the smaller the bilayer defect fraction. However, in the case of DPPA, this dependence is much more pronounced when compared to POPA and SAPA. This suggests that while all the PAs have an effect on membrane organization, the effect is, in general, the strongest in the case of unsaturated PA species.

The effect of PA lipids on the cholesterol membrane suggests a sort of specific interaction with DPPA. Both MT and the lateral (2D) diffusion coefficient were, unsurprisingly, higher when PA was added. However, when other parameters are taken into account, an interesting tendency can be observed. Tilt modulus was lower for all PA lipid species, but it was the lowest for DPPA. This suggests a strong effect of DPPA and cholesterol on the orientation of the molecules and/or curvature of the membrane. It may also be related to reorientation of the lipids in the membrane due to phase transition and formation of microdomains. Snapshots of DPPA and Chol/DPPA systems are presented in Figure 1A,B for visualization of this phenomenon. Interdigitation was similar in the case of both pure PA and Chol/PA systems with the exception of DPPA, for which it was significantly lower.

Finally, the decrease in K_A_ value was the smallest in the case of DPPA, and stronger for POPA and SAPA systems. It is worth underlining that this may be caused, again, by a difference in the bilayer phase state. The results of defect analysis in the case of Chol/PA membranes were quite complex to interpret (see Figure 2B). Firstly, pure cholesterol membrane has the highest bilayer defect fraction when compared to systems with Chol/PA. This situation, however, changed when the radius of the sphere probe increased to 0.3 nm. Interestingly, at this point for both Chol/POPA and Chol/SAPA, the bilayer defect fraction becomes higher than in the case of Chol. Furthermore, the fraction remains the lowest for the Chol/DPPA system. This suggests that the strongest effect, although potentially unfavorable for protein–lipid interactions, is observed for bilayers composed of DPPA and Chol.

Finally, the effect of PA on the membrane system with both POPC and cholesterol was assessed. MT was, in general, not affected by the presence of PA in the POPC/Chol membrane, nor was the tilt modulus with the exception of the DPPA membrane. This was similar to the effect observed in the case of DPPA/Chol, which suggests a strong interaction between these two species. The lateral diffusion coefficient was increased significantly as a result of PA addition/presence, and the highest values were observed in the case of POPA. Interestingly, such an effect was not observed for POPC/POPA or for Chol/POPA, which points to some synergistic phenomena occurring within ternary lipid mixtures. Interdigitation was affected neither in the presence of POPA nor DPPA; however, its value was significantly increased for SAPA, similarly as in the case of POPC membranes. Finally, K_A_ was in general, higher when PA was present in the membrane. Unsurprisingly, it was the highest in the case of DPPA membrane, again most likely due to the gel state of the bilayer. The defect analysis for three-component systems is in agreement with results obtained for two-component systems (see Figure 2C). Specifically, the highest bilayer defect fraction can be observed in the case of the POPC/Chol system. The presence of PA in the membrane system decreased this fraction. In this regard, the difference between POPA and SAPA was indistinguishable up to 0.4 nm probe radius. Remarkably, this border value corresponded well to the diameters of amphipathic helixes (approx. 0.85 nm) [16,21], which hence are the sizes most relevant to study the protein–membrane interactions. The DPPA effect is stronger than for unsaturated species, i.e. POPA and SAPA. Finally, each POPC/Chol/PA system has a higher bilayer defect fraction up to 0.40 nm probe radius, but the POPC/Chol/DPPA defect fraction is the lowest for the higher probe radius. Results for the simple exposed acyl chain region F_exp_ are presented in the Supporting Information (Section S1.3) along with the results for the function of probe size for homogeneous systems. These results showed that, apart from the diffusion coefficient and interdigitation, POPC/Chol/POPA and POPC/Chol/SAPA membranes are very similar.

### 2.2. Monolayers

Langmuir monolayer isotherms were the source of experimental verification of selected parameters obtained from simulated MD systems. One of the most useful applications of Langmuir monolayers is the determination of compression isotherms. The lipid monolayer, which is located at the air/liquid interphase, is compressed at a constant temperature, while the surface pressure is recorded as a function of the area available to each molecule. This approach not only allows to determine the average area of lipid molecules but may also provide information about phases and domain formation within the investigated system. Such behavior of monolayers depends mainly on the physical and chemical properties of the investigated molecules and temperature, composition, and pH of the subphase. This approach was used to precisely determine APL, surface elasticity and excess free energy of mixing of investigated model membrane systems. APL of a bilayer, or even monolayer, provides information about the 2D density. This parameter is highly sensitive to the net force of forces exerted by head groups and non-polar hydrocarbon tails. Additionally, interactions of the head groups with surrounding components, such as ions, or even water molecules, additionally influence this parameter. The results of our measurements are presented in Table 2, with a more detailed isotherm visualization presented in Supporting Information (Section S2.1). Most of the observed differences in APL data between the studied monolayers were statistically significant. A small fraction was not; however, this was not surprising taking into account the expected similarity between the APL of the investigated systems. In the case of one-component lipid monolayers, particularly interesting was the lack of statistical difference between POPA and SAPA systems as well as between Chol and DPPA systems. More detailed information about statistical significance is presented in Supporting Information (Section S2.2). In the case of two-component systems, the interesting lack of statistically significant difference was observed between POPC/POPA, Chol/POPA, Chol/SAPA, and single component monolayers of Chol or DPPA. Nevertheless, our data at least in the case of some lipid systems show a high degree of consistency with already published values. APL of POPA monolayers was previously reported to be around 60 Å^2^ when the subphase pH was equal to 8 or 7.2 and around 50 Å^2^ at pH 5 at surface pressure 30 mN/m [22,23]. In our study, we observed that APL of the POPA monolayer at pH 7.5 was equal to $53.6\pm 2.3\;{Å}^{2}$. Similarly, the value for the POPC/Chol/POPA system fits well within the histogram obtained in molecular dynamics simulations by Cheng et al. [24]. Furthermore, in our study, we obtained the APL value of the DPPA monolayer equal to $39.5\pm 1.3\;{Å}^{2}$. This value is at the lower edge of the range of DPPA APL reported in the literature, i.e., between 40 and 42 Å^2^ at 20–25 °C and subphase pH varying from 6 up to 8 [25,26,27,28,29]. Finally, the APL obtained for both POPC and cholesterol monolayers also corresponds to already published data [30,31]. Additionally, the APL value for the POPC/Chol system was $47.1\pm 1.1\;{Å}^{2}$, which is similar to what others reported [32]. Taken together, we gained confidence regarding the data reproductivity of our experimental system and, most importantly, we were able to fill gaps and obtain a more complete picture of the behavior of different PA species in two- and three-component lipid monolayers.

**Figure 3:**
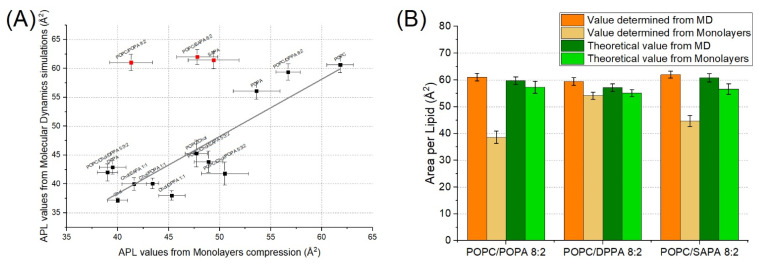
(**A**) Linear correlation of APL values from monolayer and molecular dynamics systems. (**B**) Effect of PA on POPC membranes showed with determined values of APL for POPC/PA systems for both simulation and experimental approaches. Theoretical values were determined based on values from single-component systems multiplied by factor corresponding to molar concentration on the membrane (Equation (3)).

According to various studies at surface pressure around 30–33 mN/m the area per lipid in a monolayer system corresponds to that of a bilayer [33,34,35,36]. This allows for direct comparison between membrane molecular dynamic systems and experimental Langmuir monolayers. The values obtained with these two approaches remain consistent/correlated with an R^2^ value of 74% (as shown in Figure 3A); however, three lipid mixtures are outside of this correlation. The first two, POPC/POPA and POPC/SAPA, are not surprising. Firstly, we have previously found with our monolayer results that both POPA and SAPA lipids have a strong affinity towards POPC lipids. Furthermore, it was suggested that PAs form complexes with PC [28]. This complex strongly depends on the composition of the membrane and acyl chain type. Such changes could result in an increase in packing in PA domains, as shown in the case of DPPA [37]. However, this does not fully explain the observed magnitude of such interactions. The third system was surprising, as it was a simple SAPA monolayer. In this system, no complex interaction between different lipid species could take place. The reason for such a discrepancy may lie in long-range Lennard-Jones interactions. Yalun et al. [38,39] found that depending on the cut-off distance the difference between the experimental monolayer and simulation monolayer surface area per lipid can be significant. It was also verified whether the difference between monolayers and MD studies originate from a temperature difference between the systems. Additional simulation of the POPC/POPA system was carried out at 22 °C, but no such effect was observed [data not shown]. Therefore, it could be reasonably assumed that this effect is stronger for lipids with longer acyl chains. Furthermore, the high degree of unsaturation of SAPA could be a possible factor as well.

**Figure 4:**
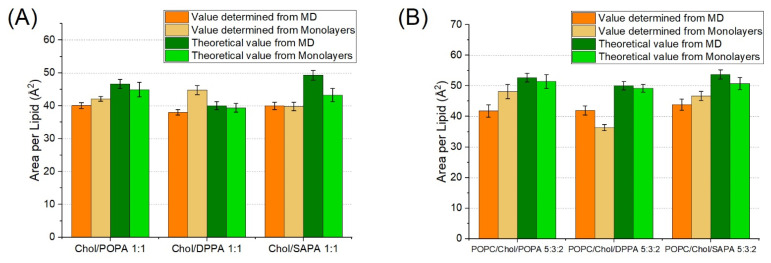
(**A**) Effect of PA on Chol membranes and (**B**) effect of PA on POPC/Chol membranes in APL determination for both simulation and experimental approaches. Theoretical values are determined based on values from single-component systems multiplied by a factor corresponding to molar concentration on the membrane (Equation (3)).

As shown in Figure 3B, APL values of both POPA and SAPA together with POPC determined by experimental monolayer isotherms are significantly lower than theoretically calculated APL using both simulation and averaged experimental values from single-component counterparts. On the other hand, the values obtained for POPC/DPPA are in agreement with the theoretical results. This strengthens the hypothesis of interaction between unsaturated species of PA and POPC within membranes. The trend observed for cholesterol and PAs is depicted in Figure 4A. The value of APL determined for the Chol/DPPA monolayer is significantly higher than the theoretical values and the value from molecular dynamics. Such an effect was observed neither for POPA nor for SAPA lipid species. Finally, for three-component systems, APL values determined using both molecular dynamics and Langmuir monolayers are, generally, slightly lower than theoretically calculated, as presented in Figure 4B. This is especially strongly visible in the case of DPPA, but weakly in the case of POPA (yet still statistically significant). This is particularly interesting because for POPA and SAPA the decreasing effect was strong in POPC/PA membrane while for DPPA the increasing effect was observed in the case of Chol/PA membrane. Such results suggest that the interaction between the particular pairs of molecular species is, to some degree, limited, due to the presence of another lipid membrane component.

The Langmuir monolayers also allow the determination of compressibility modulus C_s_, alternatively known as ‘surface elasticity’. It provides information about the physical resistivity of the studied lipid monolayer system during the compression. As a result, it strongly depends on the speed of the monolayer compression, and hence cannot be directly correlated with K_A_ from MD. The presented results are in agreement with literature data for POPC and cholesterol [30], taking into account the two times lower compression rate in our experiments. Furthermore, we observed a slight decrease in compressibility for POPC/Chol, while in the literature it was stated that there should be no change [32]. When investigating the compressibility modulus of one component monolayers there was no difference between the POPC, POPA, and SAPA systems. The DPPA system compressibility modulus was higher, which is unsurprising due to DPPA being in the gel state. It was also higher than reported in the literature data [28], which can be easily explained by the higher compression rate than in our experiments. An interesting effect can be observed when investigating the system with both POPC and PA. Specifically, a decrease was observed for POPC/POPA and POPC/SAPA systems compared to the pure POPC monolayer (stronger for the former). Additionally, the compressibility of the POPC/DPPA system was mildly higher than the one of the POPC, which is surprising as DPPA being in the gel state should stiffen the monolayer. A similar effect was observed in the case of Chol/PA systems, but it should be noted that there were higher quantities of PA in these systems. The obtained compressibility was lower than could be expected when taking into consideration individual lipid components (from the theoretically calculated value based on single monolayer systems). Most interestingly, Chol/DPPA monolayer compressibility was equal to that of the pure cholesterol system. It is possible that the change in orientation of acyl chains that was observed in MD simulation led to a change in water density near DPPA head groups, thus changing its behavior and making it less resistant. It is also possible that microdomains formed in this system (which is manifested as kinks on isotherms) triggered such behavior. Finally, in three-component systems for both POPA and SAPA, a slight stiffening effect of cholesterol was observed when compared to the corresponding systems without cholesterol. Interestingly, the compressibility of POPC/Chol/DPPA was lower than in the corresponding POPC/DPPA system (which is in agreement with the effect observed in the case of the Chol/DPPA system).

**Figure 5:**
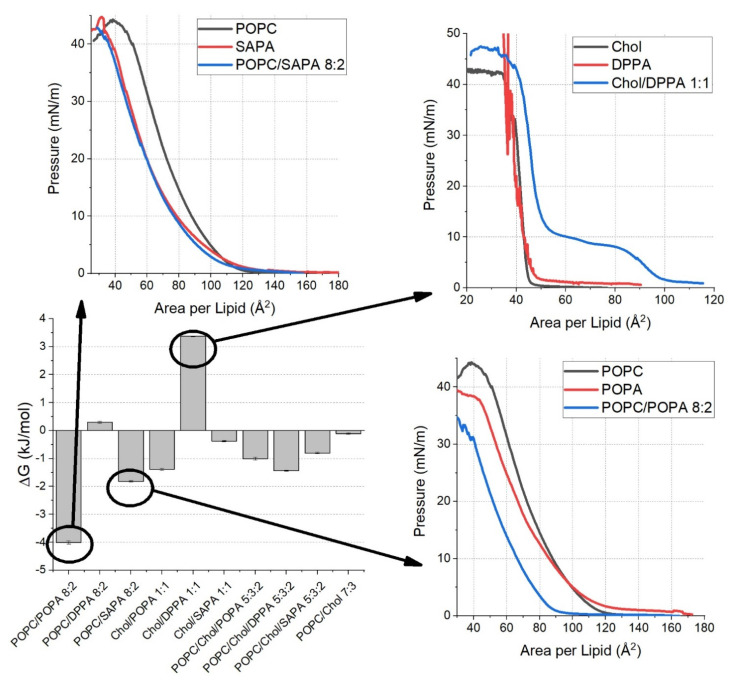
Excess free energy of mixing for investigated membrane systems with insets showing mixing of the most interesting two-component monolayers—POPC/POPA, POPC/SAPA, and Chol/DPPA. The rest of the mixing is presented in Supporting Information (Section S2.3).

The thermodynamic characteristics of mixed monolayer systems can be examined with Gibb’s free energy of mixing [29]. This term consists of two components—ideal free energy of mixing and excess free energy of mixing ΔG_m_^ex^. The latter parameter represents the deviation of free energy of mixing. In the case of an ideal miscible system, ΔG_m_^ex^ should equal zero. To this end, ΔG_m_^ex^ was calculated for the investigated monolayer systems. The values are presented in Figure 5 for isotherms up to 30 mN/m pressure with visualization of mixing of selected systems. The mixing behavior of the other systems is presented in Supporting Information (Section S2.3). Reported ΔG_m_^ex^ values for each of the lipid mixtures tested were significantly different. The data for POPC/Chol are in agreement with the literature [30] as regards the trend, i.e., the energy of mixing decreases with increasing pressure. The three most outstanding cases are for the POPC/POPA, POPC/SAPA, and Chol/DPPA system, and corresponding values of ΔG were either very low ($-4.01\pm 0.06)\;\frac{\mathrm{kJ}}{\mathrm{mol}}$, low $\left(-1.82\pm 0.03\right)\frac{\mathrm{kJ}}{\mathrm{mol}}$ or very high $\left(3.37\pm 0.02\right)\frac{\mathrm{kJ}}{\mathrm{mol}}$, respectively. In similar research [28] where DPPC/DPPA, DOPC/DPPA, and DOPC/DOPA systems were studied, it was found that a difference between the saturation in acyl chains and/or length of acyl chains between the membrane components influenced ΔG_m_^ex^. PA acyl chains shorter and/or more saturated in comparison to compared to PC present in the studied membranes resulted in positive ΔG_m_^ex^. When acyl chain length was similar, ΔG_m_^ex^ of the unsaturated (DOPC/DOPA) system was close to 0, but it was negative for the saturated (DPPA/DPPC) system. The POPC/POPA membrane system studied by us is a specific one—one acyl chain has 16 carbons, while the other has 18 carbons and one double bond. It is possible that those two aspects result in significantly lower ΔG_m_^ex^ compared to other systems. On the other hand, it is possible that such acyl chain configuration results in certain freedom of the headgroup of the lipid and allows for stronger interaction between the PC/PA that is not so easily achievable in other systems. Similar phenomena could be observed in the case of POPC/SAPA, but the saturation of acyl chains may be a more crucial factor here. The effect observed for Chol/DPPA may be due to the cholesterol condensing effect [40] on lipids in the gel phase—a decrease in membrane thickness results in an increase in area per lipid. This could result in higher ΔG_m_^ex^ of the mixture than individual single component systems. However, it is possible that reorganization of the lipid orientation, as seen in MD studies, results in the heterogeneity of phases. Such heterogeneity could result in interdigitated regions similar to those observed in the DMPC rippled phase [41]. This could explain the initial bump in the Chol/DPPA isotherm. The other interesting aspect of ΔG_m_^ex^ measurements is that both POPC:Chol:POPA and POPC:Chol:SAPA systems have lower mixing energy than their counterparts with POPC:PA. This could suggest that cholesterol plays an important role in ordering PC and PA lipids, although not predominant for POPA and SAPA lipid species, which is in agreement with the conclusions from APL.

### 2.3. Bending Rigidity Determination Using Flicker Noise Spectroscopy

**Figure 6:**
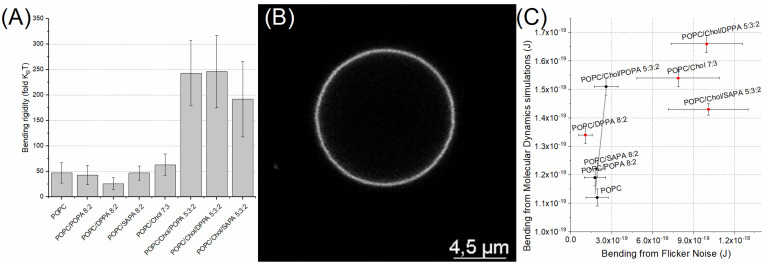
(**A**) Bending rigidity for investigated systems determined using statistical approach. (**B**) Example of recorded POPC/Chol/SAPA vesicle. (**C**) Correlation of bending rigidity values from flicker noise spectroscopy and molecular dynamic systems.

Flicker noise spectroscopy was employed to experimentally confirm bending rigidities determined using MD studies. This is necessary because the real-space fluctuation (RSF) method used for bending rigidity calculation in MD does not always reflect accurately the real membrane system. Therefore, measurements were performed on giant unilamellar vesicles (GUVs) of selected (when the formation of vesicles was achievable) membrane systems to determine their bending rigidity. In this technique, GUVs were used to allow the microscopical observations of the fluctuations of membranes, which enabled us to determine mechanical parameters. The results of these measurements are presented in both Table 2 and Figure 6A. An example snapshot of a GUV is presented in Figure 6B. The results were statistically significant. Detailed information about results from both methods as well as sample snapshots from recorded GUVs are presented in Supporting Information (Section S3). The correlation between bending rigidity obtained from simulation and experimental results is presented in Figure 6C. The values are in agreement with an R^2^ value equal to 81%. It should be noted that values obtained from flicker noise spectroscopy are in general higher than those obtained from molecular dynamics. Even in the simple POPC system, the values differ with POPC bending equal to $\left(1.93\pm 0.8\right)\times 10^{-19}\;J$ from flicker technique and $\left(1.12\pm 0.03\right)\times 10^{-19}\;J$ from MD. It was recently explained by Faizi et al. [42] that a larger pinhole size (1 Airy unit) results in a slightly increased value of bending rigidity and higher deviations. However, it should be noted that as long as membrane systems with the same pinhole size were compared, this is not an issue. Furthermore, it was observed that over 2–3 min of laser exposure some vesicles with 2 mol% dye concentration developed inward structures such as buds or visible tubes. That contributes to significantly higher bending rigidity. However, when possible, this issue was limited by vesicle selection and the statistical approach to the measurement. In our correlation analysis, four outliers can be seen. The first one, the POPC/DPPA membrane, can be easily explained by the difference between results from flicker noise spectroscopy and real space analysis that was covered elsewhere [43]. The issue with POPC/Chol/PA systems is, unfortunately, of a more complex nature. A similar issue was recently considered by Nagle et al. [44]. Namely, a difference between bending of DOPC/Chol membrane obtained from X-ray diffuse scattering and neutron spin echo and NMR relaxation is addressed. The conclusion being that both techniques measure different states of equilibration of membrane systems. Similarly, in our study, it can be argued that due to the time span of the measurement the flicker noise approach gives information about dynamic fluctuation while molecular dynamics, being an equilibrated system with a substantially shorter fluctuation collection period, focus on non-dynamic time-averaged fluctuations. As a result, a difference in bending rigidity is observed between POPC/PA systems and POPC/Chol/PA systems with flicker noise while, at the same time, such a difference was not observed in real space analysis performed on MD systems.

Based on our results it can be concluded that neither POPA nor SAPA significantly altered the mechanical behavior of the POPC membrane. However, a slight decrease in bending rigidity was observed in the case of experimental measurement of the POPC/DPPA membrane. Investigation of Chol/PA systems was experimentally limited, because both molecules, due to the packing parameter of their molecules, are unable to form GUVs. To this end, only simulation data can be analyzed. However, no difference was observed between Chol/PA systems; they were lower than the one for pure Chol and higher than for corresponding pure PA systems. Unsurprisingly, cholesterol had a statistically significant stiffening effect on the POPC membrane. Furthermore, values obtained for the POPC/Chol membrane were significantly different among all PC/PA systems. Interestingly, a strong effect of cholesterol was observed in systems with both POPC and PA, which was not visible in MD studies. For instance, the POPC/POPA membrane bending rigidity increased 5-fold when cholesterol was added to the membrane. Similarly, although with a lower amplitude, an effect was observed in the case of POPC/Chol/DPPA membrane and in POPC/Chol/SAPA membrane. This phenomenon cannot be explained by the simple effect of cholesterol, as in POPC/Chol values of bending rigidity were lower. It suggests a strong synergistic effect of both PA and Chol on membrane mechanics, which does not occur in the case of two-component membranes. Interestingly, experimentally determined values of bending rigidity of POPC/Chol/DPPA and POPC/Chol/POPA membranes were not statistically different.

### 2.4. Interactions of PA Lipids with PC and/or Cholesterol Wrapped Up in a Summary

Both POPA and SAPA showed strong interaction with POPC membranes. This was especially visible in APL changes. Additionally, strong interactions occur between POPC and POPA or SAPA, as indicated by negative values of ΔG_m_^ex^. Interestingly, K_A_ increased for POPA but greatly decreased for SAPA compared to the pure POPC system, which suggests an additional/stronger effect of SAPA likely related to acyl chain length and/or saturation. Both POPC/POPA and POPC/SAPA APL were lower than in their pure counterparts which suggests a strong interaction between the PC/PA head groups (membrane thickness of PA species in membranes with POPC were similar). However, no such interaction was observed for DPPA. On the other hand, DPPA was the only PA lipid that influenced the mechanical changes of the membrane (specifically, bending rigidity decreased compared to POPC membrane). This, however, may be more related to the different phases of DPPA than the effect of POPC/DPPA interactions. When the membrane defects are analyzed all of the PAs exert very similar effects which are reflected by the PA-induced increase in the exposed acyl chain region F_exp_. This is additionally confirmed when the defects are probed with spheres of radius 0.25 nm—each POPC/PA system has a higher bilayer defect fraction than the pure POPC system. Interestingly, when single systems are taken into account the highest bilayer defect fraction is observed for SAPA, which is followed by POPA. However, such a difference was not observed for POPC/SAPA and POPC/POPA systems.

Contrary to POPA and SAPA, DPPA exerts a strong effect on cholesterol. It is strongly visible in APL changes in monolayer systems (the APL of Chol/DPPA is higher than these of Chol and DPPA systems individually). The high negative value of ΔG_m_^ex^ additionally confirms that. Interestingly, this effect was not observed in the case of the simulated Chol/DPPA system, which suggests that this relation is based on interactions not covered by the molecular dynamic system and/or this phenomenon is of a longer timescale. On the other hand, a difference was observed in interdigitation, since in the Chol/DPPA system it was weaker than in either the Chol or DPPA system. Additionally, a strong shift in lipid orientation was observed, which is reflected by the reduction in tilt modulus. For bending rigidity analyzed via molecular dynamics, the value was lower for Chol/DPPA than for corresponding single-component systems. In defect analysis, interestingly, cholesterol increased the fraction of exposed acyl chains with DPPA. This was opposite to both POPA and SAPA, where a decrease occurred (when compared to single PA systems). Furthermore, in MD studies a strong reorganization of acyl chain orientation was observed in the Chol/DPPA system compared to DPPA. Taking together, the data collected for APL changes, bending changes, defect analysis and interdigitation suggest that cholesterol and DPPA strongly interact with each other, which dramatically changes membrane organization. It also should be noted that Chol, in general, had a strong condensing effect on all of the investigated systems. Namely, the K_A_ value was higher in the case of all the systems with Chol than in respective systems without Chol, though this effect was the strongest in systems with DPPA. It seems that the presence of Chol contributed mostly to the compressibility, as its effect on bending rigidity was not so strong. However, it should be noted that the increase in bending rigidity in the case of the POPC/Chol 7:3 membrane system was reported to be around 40% [45].

When three-component systems were considered the most peculiar phenomenon was observed with mechanical properties. Specifically, the bending rigidity increased 4- (for POPA and SAPA) and 8-fold (for DPPA) compared to systems without cholesterol. This effect cannot be influenced only by stiffening due to the presence of cholesterol, as in the corresponding POPC/Chol such an increase was not observed. A slight increase in membrane thickness was observed in all PA cases compared to POPC. Interestingly, interdigitation did increase in the case of SAPA and POPA, but not DPPA. Area per lipid of PA species always decreased in systems with cholesterol with the exception of the POPC/Chol/DPPA system, where it was higher compared to the single DPPA system. Furthermore, the compressibility of each of the POPC/Chol/PA membranes was higher than both POPC/Chol and respective POPC/PA systems. This suggests that the presence of cholesterol influenced the membranes with PA in the acyl chain regions. Interestingly, there was no specificity with POPC/DPPA membrane that was visible when two-component systems were analyzed. There was no substantial change after the addition of cholesterol to POPC/PA membranes, although a slight decrease of the polar surface fraction was observed.

## 3. Materials and Methods

### 3.1. Materials

Lipids POPC (1-palmitoyl-2-oleoyl-glycero-3-phosphocholine), POPA (1-palmitoyl-2-oleoyl-*sn*-glycero-3-phosphate), DPPA (1,2-dipalmitoyl-*sn*-glycero-3-phosphat), SAPA (1-stearoyl-2-arachidonoyl-*sn*-glycero-3-phosphate), cholesterol and 16:0 Liss Rhod PE were purchased from Avanti Polar Lipids (Alabaster, AL, USA). The fluorescent probe Atto488-DOPE was purchased from Atto-Tech (Siegen, Germany). HEPES was purchased from Carl Roth (Karsruhe, Germany). The ultra-pure water used in experiments was obtained from a water purification system (Millipore, Burlington, MA, USA).

### 3.2. Molecular Dynamics (MD) Simulations

The full-atomistic MD simulation was performed using NAMD 2.13 [46] software with CHARMM36 force fields [47] under NPT conditions (constant: number of particles, pressure, and temperature). Lipid membrane systems consisted of 648 lipid molecules (324 on each of leaflets). The system was hydrated in such a way that 75 water molecules per lipid molecule were used. All systems were additionally neutralized with positive counter ions. All systems were equilibrated using a standard equilibration procedure. Total simulation time for all systems was at least 30 ns with the last 10 ns used for analysis. Simulations were carried out at 30 °C (303.15 K).

### 3.3. MD System Characteristics

**Area per Lipid and Membrane Thickness.** Both membrane area per lipid (APL) and membrane thickness (MT) were determined using a custom Matlab script. The midpoint position between P and C2 atoms was used as a point position for each lipid. This was followed by Voronoi tessellation to obtain the individual APL for each lipid molecule in every simulation time step. The APL value for the whole membrane is determined by Gaussian fitting to histogrammed APL values of all molecules. The APL value for a given type of molecule is determined by averaging. MT was calculated as the difference between mean height values of atoms in opposite leaflets. The parameter was determined for P, C1, and C2 atoms. For cholesterol corresponding C3, C2, and C1 atoms were selected. Both APL and MT were calculated for the whole system and for individual lipid species in the system. Appropriate atoms are indicated in Supporting Information Figure S2.

**Bending Rigidity and Tilt Rigidity.** To determine mechanical parameters (focusing on bending rigidity coefficient, but also tilt modulus) a real-space fluctuation (RSF) method was used [48]. Specifically, a probability distribution for both tilt and splay is determined for all lipids over all analyzed time steps. Tilt θ is defined as the angle between the lipid director and bilayer normal. Lipid director is defined as the vector between the lipid head point (midpoint between C2 and P atoms) and the lipid tail point (midpoint between last carbon atoms). Lipid splay S_r_ is defined as the divergence of the angle formed by the directors of neighboring lipids providing that they are weakly correlated.

**Compressibility.** A method developed by Doktorova et al. [49] was used to determine the compressibility modulus of lipid membranes. Briefly, fluctuations of membrane thickness t, rather than membrane area fluctuations, are used as a means to determine the leaflet compressibility modulus K_A_. Using both thermodynamic and statistical approaches an Equation (1) can be determined, which is directly used to determine K_A_. In Equation (1) a_0_—area per lipid, t_0_—equilibrium membrane thickness, t—instantaneous membrane thickness, k_B_—Boltzmann constant, and T—temperature. In this method, first, the optimal membrane thickness layer is determined to avoid both over calculation and under calculation of K_A_. This is followed by local fluctuation binning to determine the potential of mean force (PMF). Relative changes of membrane thickness are used to determine K_A_. Originally, the method was used to determine K_A_ for individual membrane leaflets, but recent literature suggests that the value is indeed for the whole membrane [50].

(1) $$-\frac{2k_{B}T}{a_{0}^{L}}\ln p\left(\frac{t_{0}^{L}-t^{L}}{t^{L}}\right)=K_{A}^{L}\left(\frac{t_{0}^{L}-t^{L}}{t^{L}}\right)+C^{\prime }$$

**Lateral Diffusion Coefficient.** The Diffusion Coefficient Tool plugin was used for the determination of lipid molecules’ lateral (2D) diffusion [51]. It is calculated using Einstein’s relation with mean square displacement (MSD) of the chosen molecular species.

**Interdigitation.** The interdigitation parameter was determined using MEMBPLUGIN [52]. The obtained parameter is defined as the width of the region of mass overlap.

**Acyl chain accessibility.** A protocol implemented by Boyd et al. [19] was used for acyl chain accessibility determination. Briefly, the occupancy of each bilayer atom (from lowest to highest) is calculated based on Van der Waals radius. If it is a headgroup atom the sphere is marked as polar, otherwise as a tail. The headgroup is defined as all atoms down to and including the C2 atom. This is followed by dividing the simulation box into square grids with a grid spacing of 0.5 Å. For each grid position, a value of the occupancy graph is checked. As a result, a 2D map of either the head or tail region is obtained, which is used to determine the fraction of the bilayer surface allowing access to the acyl chain region. This is followed by sphere probe analysis in order to ignore small gaps in headgroup coverage. For each point, a sphere probe of radius ranging from 0.1 nm up to 0.5 nm is set. If all the grid points in the range of this probe are classified as tails, those grid points are assigned as defects. In this way, only hydrophobic patches with given sizes and shapes are classified as true defects.

### 3.4. Surface Pressure–Area (π–A) Isotherm Measurement

The π–A isotherms of all monolayers were measured using a computer-controlled Langmuir-type film system (Kibron). Before measurement, the trough (205 × 60 mm^2^) was carefully cleaned with water and ethanol and dried. This was followed by filling the trough with 5 mM HEPES/KOH (pH 7.4) as the aqueous subphase. Lipid solution in hexane was spread over the subphase between barriers. The concentration of lipids was determined using the total phosphorus assay method [53]. After the organic solvent was completely evaporated (about 10 min.), the monolayer was compressed at a rate of 10 mm/min. The compression was carried out with two sliding barriers moving with the same speed from the edge of each compartment to its center, where the Wilhelmy plate, used as a surface pressure sensor, was placed. The subphase temperature was kept at 22.0 ± 0.5 °C. The measurements under the same conditions were independently repeated to obtain at least five π–A isotherm profiles. A monolayer was compressed until the surface pressure was 3 mN/m lower than the collapse pressure (which was determined during the first run).

### 3.5. Surface Pressure–Area (π–A) Isotherm Analysis

APL of the investigated monolayers was collected at a pressure of 33 mN/m. The area compressibility was calculated according to Equation (2), where A is the area per molecule at the indicated surface pressure and π is the corresponding surface pressure [54].

(2) $$C_{s}=\left(-\frac{1}{A}\right)\frac{\mathrm{dA}}{d\pi }$$

The excess free energy of mixing (${\Delta G}_{m}^{\mathrm{ex}}$) was calculated for π–A isotherms for pure and mixed monolayers following the established protocols [55,56,57]. The values were calculated in the range from 0 to 33 mN/m. Briefly, the theoretical mean APL for non-interacting molecules was calculated as in Equation (3), where A_i_—the mean molecular area, X_1_, X_2_—mole fraction of component 1 or 2, A_1_ and A_2_—mean molecular areas of pure components 1 or 2. If three-component monolayers were analysed an additional compartment was added.

(3) $$A_{i}=X_{1}A_{1}+X_{2}A_{2}$$

The excess free energy of mixing was calculated according to Equation (4) (in the case of two-compartment monolayers) and Equation (5) (in the case of three-component monolayers), where N_A_—Avogadro’s number. Calculations were carried out using a custom MATLAB script. The deviation was calculated as the sum of deviations of all involved APL at a given pressure value.

(4) $$\Delta G_{m}^{\mathrm{ex}}=N_{A}\left(\int_{\pi_{0}}^{\pi }A_{12}d\pi -x_{1}\int_{\pi_{0}}^{\pi }A_{1}d\pi -x_{2}\int_{\pi_{0}}^{\pi }A_{2}d\pi \right)$$

(5) $$\Delta G_{m}^{\mathrm{ex}}=N_{A}\left(\int_{\pi_{0}}^{\pi }A_{123}d\pi -x_{1}\int_{\pi_{0}}^{\pi }A_{1}d\pi -x_{2}\int_{\pi_{0}}^{\pi }A_{2}d\pi -x_{3}\int_{\pi_{0}}^{\pi }A_{3}d\pi \right)$$

### 3.6. GUVs Electroformation

The modified method of formation for giant unilamellar vesicles (GUVs) was used. Briefly, 10 µL of a 1 mM POPC and fluorescent probe mixture (1 m%) in chloroform was distributed equally along the platinum electrodes and dried under reduced pressure for 1 h. The electrodes were then submerged in aqueous 5 mM HEPES/KOH (pH 7.4) and a sinusoidal 10 Hz AC electric field was applied for 2 h with 1 V voltage in a custom PTFE (polytetrafluoroethylene) electro-formation chamber [58].

### 3.7. Flicker Noise Spectroscopy

Thermally-induced shape fluctuations of GUVs can be used to determine the mechanical properties (bending rigidity). The series of images of GUVs were recorded using confocal microscopy, i.e., a Stellaris 8 confocal microscope (Leica, Wetzlar, Germany) equipped with an HC PL APO 86x/1.20 water immersion objective (Leica, Wetzlar, Germany) and 1 Airy unit pinhole size. 256 × 256 pixels images were recorded with a hybrid (HyD) detector with pixel size ranging from 0.07 μm to 0.12 μm with video integration time ranging from 148 ms up to 189 ms depending on the zoom magnitude. Samples were illuminated with a white laser set at either 488 nm (emitted light was recorded from 500 up to 600 nm) for the Atto488-DOPE probe or 560 nm (emitted light was recorded from 570 up to 630 nm) for the Liss Rhod probe. The series usually consisted of 1200 images. The two-dimensional liposome images are transformed to the three-dimensional Helfrich model using both the average-based and statistical approaches. Then, the radial position of the bilayer, extracted from images, is used to construct angular autocorrelation curves. In the average-based approach autocorrelation curves are decomposed into Legendre polynomial series and are plotted as a function of fluctuation mode so the bending rigidity coefficient can be determined. In the statistical approach, autocorrelation curves are decomposed into Fourier series and a frequency histogram of amplitudes for each mode of fluctuation is calculated. The histogram is then used for the determination of the bending rigidity coefficient as described in detail elsewhere [59]. The radii of investigated vesicles ranged from 3.2 to 11.6 μm. The measurements were carried out at 22 °C.

### 3.8. Statistics

In order to test for significant difference between parameters, unless otherwise specified, the one-way ANOVA test was used with a significance level of 0.05. The Tukey test was used as a post hoc test. All statistical analysis was performed using the OriginPro 2018 (OriginLabs, Northampton, MA, USA) software. Average values are presented with the standard deviation.

## 4. Conclusions

In this study, we focused on the systematic characterization of membrane systems with biologically relevant PA lipid species, namely POPA, SAPA, and DPPA. These lipids were chosen due to their role in major cellular metabolic pathways the activation of which appeared to be crucial in a broad range of physiological events. PA is a precursor for the majority of cellular glycerophospholipids, acts as a signaling molecule and, due to its molecular shape, may also modulate membrane curvature. In addition, numerous PA-interacting proteins of variable functions and subcellular localization were identified, although the mechanisms that govern the specificity of such interactions are to a large degree unknown. Moreover, it is still not clear how various molecular species of PA contribute to a broad array of cellular processes. In order to (at least in part) fill this gap and gain a more precise view of molecular characteristics of different species of phosphatidic acid, the behavior of these lipids in the context of relatively simple membrane model systems was analyzed. Using a multidisciplinary approach combining both molecular dynamics and experimental approaches (flicker noise spectroscopy and Langmuir monolayer isotherms), we were able to systematically characterize the influence of PA on lipid bilayers. Our results showed a strong interaction between two species of PA—namely POPA and SAPA—with phosphatidylcholine species that are most abundantly represented in mammalian cells. On the other hand, our results also suggest that the DPPA interacted with cholesterol. Finally, a significant changes in mechanical behavior were observed for the PC/Chol/PA three-components membrane systems, which could not be directly deduced from the behavior of the corresponding two-component systems. These results suggest that different PA species have a different influence on the lipid membranes. Their behavior with other membrane lipids is complex, and certain phenomena can only be observed when lipids of various classes are present and cannot be treated as the superposition of a combination of simpler systems. In the literature, various factors have been proposed as modulators of lipid-protein interactions, including acyl chain length, membrane curvature, defects on the surface, and/or mechanical properties. These factors may determine the specific nature of protein–membrane interactions that are being investigated rather than propose a general tendency. The biophysical characterization performed on investigated systems is not limited to structural properties but can analyze emergent membrane properties such as mechanical behavior. Nevertheless, different proteins may have preferences towards various PA molecular species based on different mechanical and structural properties of target membranes as well as various presentations of the recognized lipids. Thus, it is highly probable that universal molecular mechanisms of protein–PA interactions will not be attainable based only on membrane characteristics. However, we believe that our work will reveal the detailed characteristics of a membrane system that could, when addressed to a particular PA-interacting molecule, help to establish such a link.

## Figures and Tables

**Table 1:** Molecular dynamics characteristics of membranes with PA. Presented parameters are calculated for all particles. Distinction for individual lipid species is presented in Table S1.

Lipid Bilayer Composition (Molar Ratios)	MT_P-P_	APL	κ	κ_tilt_	K_A_	2D Diffusion	Interdigitation
	nm	Å^2^	J	J	mN/m	µm^2^/s	Å
POPC	3.78 ± 0.04	60.7 ± 1.4	(1.12 ± 0.03) × 10^−19^	(2.86 ± 0.05) × 10^−20^	235 ± 23	7.7 ± 0.6	5.85 ± 0.43
POPA	4.10 ± 0.04	56.1 ± 1.4	(1.27 ± 0.03) × 10^−19^	(3.82 ± 0.07) × 10^−20^	846 ± 73	12.35 ± 0.12	5.09 ± 0.43
DPPA	4.64 ± 0.02	42.9 ± 1.2	(4.55 ± 0.13) × 10^−19^	(17.86 ± 0.54) × 10^−20^	4969 ± 208	1.57 ± 0.03	4.7 ± 0.3
SAPA	4.99 ± 0.03	61.5 ± 1.5	(1.23 ± 0.04) × 10^−19^	(3.33 ± 0.05) × 10^−20^	201.5 ± 23	9.65 ± 0.15	6.3 ± 0.5
Chol	3.18 ± 0.08	37.19 ± 0.35	(4.34 ± 0.01) × 10^−19^	(7.78 ± 0.06) × 10^−20^	16,526 ± 453	1.12 ± 0.01	3.37 ± 0.21
POPC/POPA 8:2	3.92 ± 0.03	61.1 ± 1.4	(1.19 ± 0.03) × 10^−19^	(3.47 ± 0.06) × 10^−20^	455 ± 24	10.3 ± 0.2	6.0 ± 0.5
POPC/DPPA 8:2	3.99 ± 0.03	59.4 ± 1.4	(1.34 ± 0.03) × 10^−19^	(3.52 ± 0.06) × 10^−20^	502 ± 33	13.16 ± 0.13	5.57 ± 0.44
POPC/SAPA 8:2	3.95 ± 0.03	62.0 ± 1.3	(1.22 ± 0.03) × 10^−19^	(3.39 ± 0.05) × 10^−20^	156 ± 38	10.7 ± 0.1	6.1 ± 0.5
Chol/POPA 1:1	3.97 ± 0.01	40.1 ± 0.9	(1.60 ± 0.04) × 10^−19^	(7.27 ± 0.01) × 10^−20^	8320 ± 162	3.85 ± 0.04	3.3 ± 0.3
Chol/DPPA 1:1	4.04 ± 0.01	38.0 ± 0.8	(1.59 ± 0.04) × 10^−19^	(10.1 ± 0.1) × 10^−20^	13,898 ± 270	2.83 ± 0.03	2.9 ± 0.3
Chol/SAPA 1:1	4.13 ± 0.01	40.0 ± 1.1	(1.52 ± 0.04) × 10^−19^	(6.48 ± 0.01) × 10^−20^	8304 ± 221	2.40 ± 0.06	3.7 ± 0.3
POPC/Chol/POPA 5:3:2	4.08 ± 0.02	41.8 ± 2.0	(1.51 ± 0.03) × 10^−19^	(5.9 ± 0.1) × 10^−20^	3005 ± 110	6.44 ± 0.08	3.8 ± 0.3
POPC/Chol/DPPA 5:3:2	4.17 ± 0.02	42.0 ± 1.5	(1.66 ± 0.03) × 10^−19^	(7.4 ± 0.1) × 10^−20^	7521 ± 257	5.49 ± 0.07	3.69 ± 0.42
POPC/Chol/SAPA 5:3:2	4.12 ± 0.02	43.9 ± 1.8	(1.43 ± 0.02) × 10^−19^	(5.6 ± 0.1) × 10^−20^	3908 ± 116	5.46 ± 0.07	4.22 ± 0.41
POPC/Chol 7:3	4.14 ± 0.02	45.3 ± 2.3	(1.54 ± 0.03) × 10^−19^	(5.7 ± 0.1) × 10^−20^	1948 ± 95	2.85 ± 0.02	3.75 ± 0.33

Abbreviations: MT_P-P_—membrane thickness calculated from phosphorus-to-phosphorus atoms (or C3 atoms for cholesterol), APL—area per lipid, κ—bending rigidity, κ_tilt_—tilt modulus, K_A_—area compressibility coefficient.

**Table 2:** Parameters describing membranes determined with experimental techniques–Langmuir monolayers (at pressure equal to 33 mN/m) and flicker noise spectroscopy.

Lipid Bilayer Composition (Molar Ratios)	APL	C_s_	ΔG	Κ
	Å2	mN/m		J
POPC	59 ± 1	59 ± 10	-	(1.9 ± 0.8) × 10^−19^
POPA	50.3 ± 2.2	53 ± 7	-	-
DPPA	39.3 ± 1.3	648 ± 58	-	-
SAPA	47 ± 2	53.4 ± 2.5	-	-
Chol	39.5 ± 1.2	227 ± 84	-	-
POPC/POPA 8:2	38.6 ± 2.3	35.1 ± 7.2	−666 ± 10	(1.8 ± 0.8) × 10^−19^
POPC/DPPA 8:2	54.1 ± 1.4	68 ± 13	50 ± 5	(1.1 ± 0.5) × 10^−19^
POPC/SAPA 8:2	44.6 ± 2.1	42.8 ± 4.3	−302 ± 4	(1.9 ± 0.6) × 10^−19^
Chol/POPA 1:1	42.1 ± 0.7	102 ± 34	−230 ± 6	-
Chol/DPPA 1:1	44.8 ± 1.4	227 ± 53	560 ± 2	-
Chol/SAPA 1:1	39.8 ± 1.3	68 ± 14	−63 ± 4	-
POPC/Chol/POPA 5:3:2	48.1 ± 2.3	58.5 ± 12.2	−167 ± 8	(10.0 ± 2.6) × 10^−19^
POPC/Chol/DPPA 5:3:2	36.4 ± 1	45 ± 7	−239 ± 4	(10 ± 3) × 10^−19^
POPC/Chol/SAPA 5:3:2	46.7 ± 1.5	57.1 ± 6.3	−133 ± 5	(7.8 ± 3.1) × 10^−19^
POPC/Chol 7:3	44.3 ± 1.1	51 ± 11	−18 ± 4	(2.6 ± 0.9) × 10^−19^

Abbreviations: APL—area per lipid, Cs—monolayer compressibility, ΔG_m_^ex^—excess free energy of mixing, κ—bending rigidity.

## Data Availability

The majority of data is available in Supporting Information. The rest of the data presented in this study are available on request from the corresponding authors. The raw data are not publicly available due to the significant file size of MD trajectories and recorded videos.

## Supplementary Materials

The following are available online at https://www.mdpi.com/article/10.3390/ijms222111523/s1, Section S1: Details of molecular dynamics—molecular structures, table of detailed parameters, probe defect analysis, Section S2: Langmuir monolayers—individual surface pressure-area isotherms, statistical significance of APL, excess free energy of mixing visualizations, Section S3: flicker noise spectroscopy—detailed results for individual lipids, populations and snapshots of example vesicles.
