# Supplementary material for: Simple Does Not Mean Trivial: Behavior of Phosphatidic Acid in Lipid Mono- and Bilayers
Source: Int J Mol Sci. 2021 Oct 26;22(21):11523. doi: 10.3390/ijms222111523 (PMC8584262; doi:10.3390/ijms222111523)
Supplement: Supplementary file 1 [file ijms-22-11523-s001.zip › ijms-1391863-supplementary.pdf]

Supporting Information: Simple does not mean trivial: behavior of phosphatidic acid in lipid mono- and bilayers

Dominik Drabik[a] and Aleksander Czogalla[a]

[a] Laboratory of Cytobiochemistry, Faculty of Biotechnology, University of Wroclaw, Wroclaw, Poland;

1. Molecular Dynamics

1.1 Molecular structures and schemes

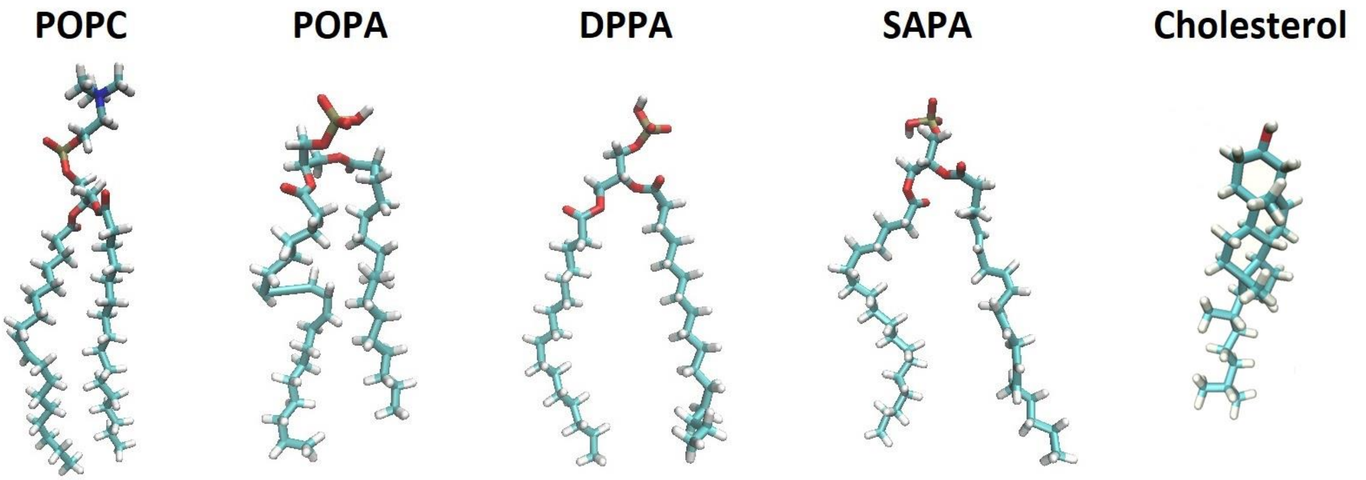

Figure S1. Structures of molecules used in this studies. From left to right are presented POPC, POPA, DPPA, SAPA and cholesterol molecules.

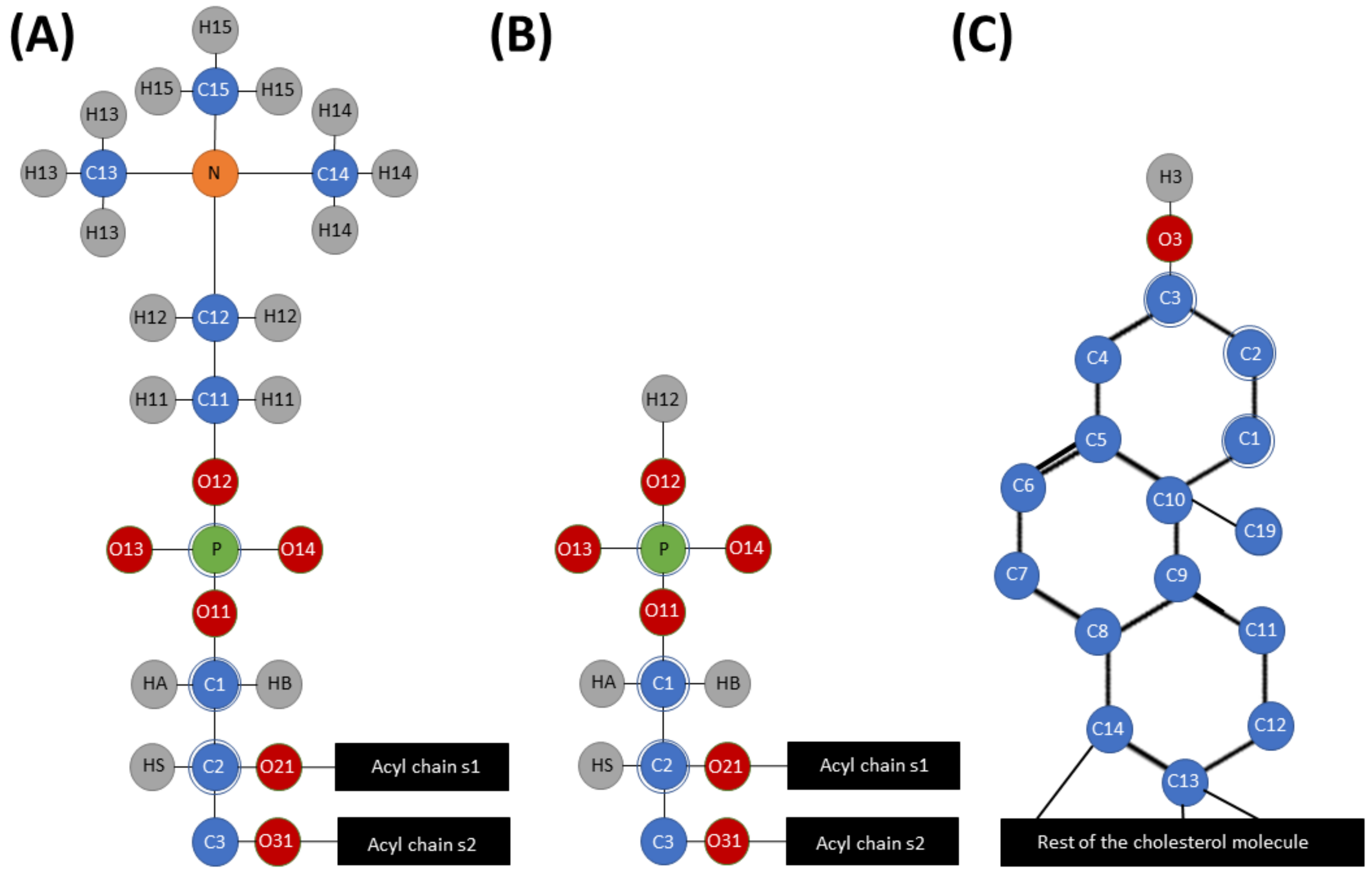

Figure S2. Schemes of molecules used in the studies with focus on individual atom names. The schemes present (A) PC and (B) PA headgroups of used lipid molecules and (C) cholesterol molecule (hydrogen atoms in carbon rings were omitted for clarity).

1.2 More detailed tables

In main text a table is with properties of whole membrane system was presented. However, some of the parameters, such as membrane thickness or area per lipid, can be calculated only for given lipid species. Furthermore, membrane thickness can be calculated based on other atoms such as C1 or C2. Additionally, total time of simulations should also be provided (for all systems last 10 ns were used for parameter determination). All this data is presented in table S1.

Tab S1. More detailed parameters determined in MD studies with distinction for individual lipid species in the membrane.

Membrane system	Lipid specie	MT_{P-P} [nm]	MT_{C1-C1} [nm]	MT_{C2-C2} [nm]	APL [nm^2]	2D diffusion [$\mu m^2/s$]	Simulation time [ns]
**POPC**	-	3,78 ± 0,04	3,39 ± 0,03	3,16 ± 0,03	60,7 ± 1,4	7,69 ± 0,56	100
**POPA**	-	4,10 ± 0,04	3,75 ± 0,03	3,52 ± 0,03	56,2 ± 1,4	12,35 ± 0,12	33,53
**DPPA**	-	4,64 ± 0,02	4,29 ± 0,02	4,05 ± 0,02	42,8 ± 1,2	1,57 ± 0,03	30
**SAPA**	-	4,99 ± 0,03	3,75 ± 0,03	3,52 ± 0,01	61,4 ± 1,5	9,65 ± 0,15	28,78
**POPC/POPA 8:2**	Total	3,18 ± 0,08	3,06 ± 0,01	2,76 ± 0,01	37,12 ± 0,32	1,12 ± 0,01	30
	POPC	3,92 ± 0,03	3,54 ± 0,03	3,30 ± 0,03	61,0 ± 1,4	10,3 ± 0,2	
	POPA	3,93 ± 0,03	3,54 ± 0,03	3,31 ± 0,03	63,6 ± 0,9	10,22 ± 0,14	
**POPC/DPPA 8:2**	Total	3,88 ± 0,04	3,51 ± 0,04	3,28 ± 0,04	61,3 ± 0,6	10,19 ± 0,06	30
	POPC	3,99 ± 0,03	3,61 ± 0,04	3,38 ± 0,04	59,3 ± 1,4	13,16 ± 0,13	
	DPPA	4,00 ± 0,03	3,60 ± 0,03	3,37 ± 0,03	61,7 ± 0,7	13,0 ± 0,2	
**POPC/SAPA 8:2**	Total	3,98 ± 0,05	3,62 ± 0,05	3,39 ± 0,05	59,1 ± 1,1	13,8 ± 0,2	30
	POPC	3,95 ± 0,03	3,56 ± 0,03	3,33 ± 0,03	62,0 ± 1,3	10,7 ± 0,1	
	SAPA	3,95 ± 0,02	3,56 ± 0,03	3,33 ± 0,02	64,4 ± 0,5	11,1 ± 0,1	
**Chol/POPA 1:1**	Total	3,94 ± 0,04	3,57 ± 0,04	3,34 ± 0,04	61,2 ± 1,2	8,93 ± 0,12	30
	Chol	3,97 ± 0,01	3,76 ± 0,01	3,50 ± 0,01	40,0 ± 0,9	3,85 ± 0,04	
	POPA	3,40 ± 0,02	3,28 ± 0,02	2,99 ± 0,02	39,0 ± 0,4	3,80 ± 0,04	
**Chol/DPPA 1:1**	Total	4,53 ± 0,02	4,23 ± 0,02	4,01 ± 0,02	45,3 ± 0,4	3,85 ± 0,06	30
	Chol	4,04 ± 0,01	3,82 ± 0,01	3,48 ± 0,01	38,2 ± 0,7	2,83 ± 0,03	
	DPPA	3,43 ± 0,02	3,30 ± 0,02	3,00 ± 0,02	37,2 ± 0,3	2,85 ± 0,05	
**Chol/SAPA 1:1**	Total	4,66 ± 0,02	4,37 ± 0,02	4,14 ± 0,02	41,6 ± 0,3	2,81 ± 0,06	34,26
	Chol	4,13 ± 0,01	3,92 ± 0,01	3,67 ± 0,02	40,1 ± 1,1	2,40 ± 0,06	
	SAPA	3,52 ± 0,02	3,40 ± 0,02	3,11 ± 0,02	39,0 ± 0,5	2,34 ± 0,06	
**POPC/Chol 7:3**	Total	4,14 ± 0,02	3,84 ± 0,02	3,84 ± 0,02	44,9 ± 2,3	2,85 ± 0,02	30,86
	POPC	4,53 ± 0,02	3,84 ± 0,02	3,84 ± 0,02	50,9 ± 0,4	2,70 ± 0,02	
	Chol	3,24 ± 0,03	3,84 ± 0,02	3,84 ± 0,02	37,7 ± 0,6	3,19 ± 0,04	
**POPC/Chol/POPA 5:3:2**	Total	4,08 ± 0,02	3,81 ± 0,02	3,56 ± 0,02	41 ± 2	6,44 ± 0,08	30
	POPC	4,53 ± 0,03	4,16 ± 0,03	3,93 ± 0,02	50,1 ± 0,5	7,25 ± 0,12	
	Chol	3,27 ± 0,03	3,15 ± 0,03	2,86 ± 0,03	39,1 ± 0,5	5,16 ± 0,03	
	POPA	4,49 ± 0,03	4,16 ± 0,03	3,92 ± 0,03	48 ± 1	6,78 ± 0,09	
**POPC/Chol/DPPA 5:3:2**	Total	4,17 ± 0,02	3,89 ± 0,02	3,64 ± 0,02	42,0 ± 1,4	5,49 ± 0,07	30
	POPC	4,64 ± 0,03	4,27 ± 0,03	4,04 ± 0,03	48,1 ± 0,5	6,10 ± 0,13	
	Chol	3,32 ± 0,03	3,19 ± 0,03	2,90 ± 0,03	36,9 ± 0,6	4,44 ± 0,03	
	DPPA	4,59 ± 0,03	4,26 ± 0,03	4,04 ± 0,03	46,1 ± 0,8	5,96 ± 0,05	
**POPC/Chol/SAPA 5:3:2**	Total	4,12 ± 0,02	3,84 ± 0,02	3,59 ± 0,02	43,9 ± 1,4	5,46 ± 0,07	32
	POPC	4,57 ± 0,03	4,20 ± 0,03	3,96 ± 0,03	50,9 ± 0,7	6,38 ± 0,08	
	Chol	3,29 ± 0,03	3,17 ± 0,03	2,88 ± 0,03	39,2 ± 0,5	4,71 ± 0,07	
	SAPA	4,57 ± 0,04	4,23 ± 0,04	3,99 ± 0,04	48,4 ± 0,7	4,69 ± 0,09	

Abbreviations: MT_{P-P} – membrane thickness determined for phosphorus atoms (C3 atoms for cholesterol); MT_{C1-C1} – membrane thickness determined for C1 carbon atoms (C2 atoms for cholesterol); MT_{C2-C2} – membrane thickness determined for C2 carbon atoms (C1 atoms for cholesterol); APL – area per lipid.

1.3 Probe defects analysis

Below are presented more detailed information about defect analysis on investigated membrane systems. The evolution of fraction polar surface in time is presented in Figure S.3 panel A. The average values and standard deviations are presented in panel B. Membrane defects fraction probed at given probe radius equal to 0.25 nm is presented in panel C. Finally, bilayer defect fraction in function of increasing probe radius for single component systems is presented in panel D.

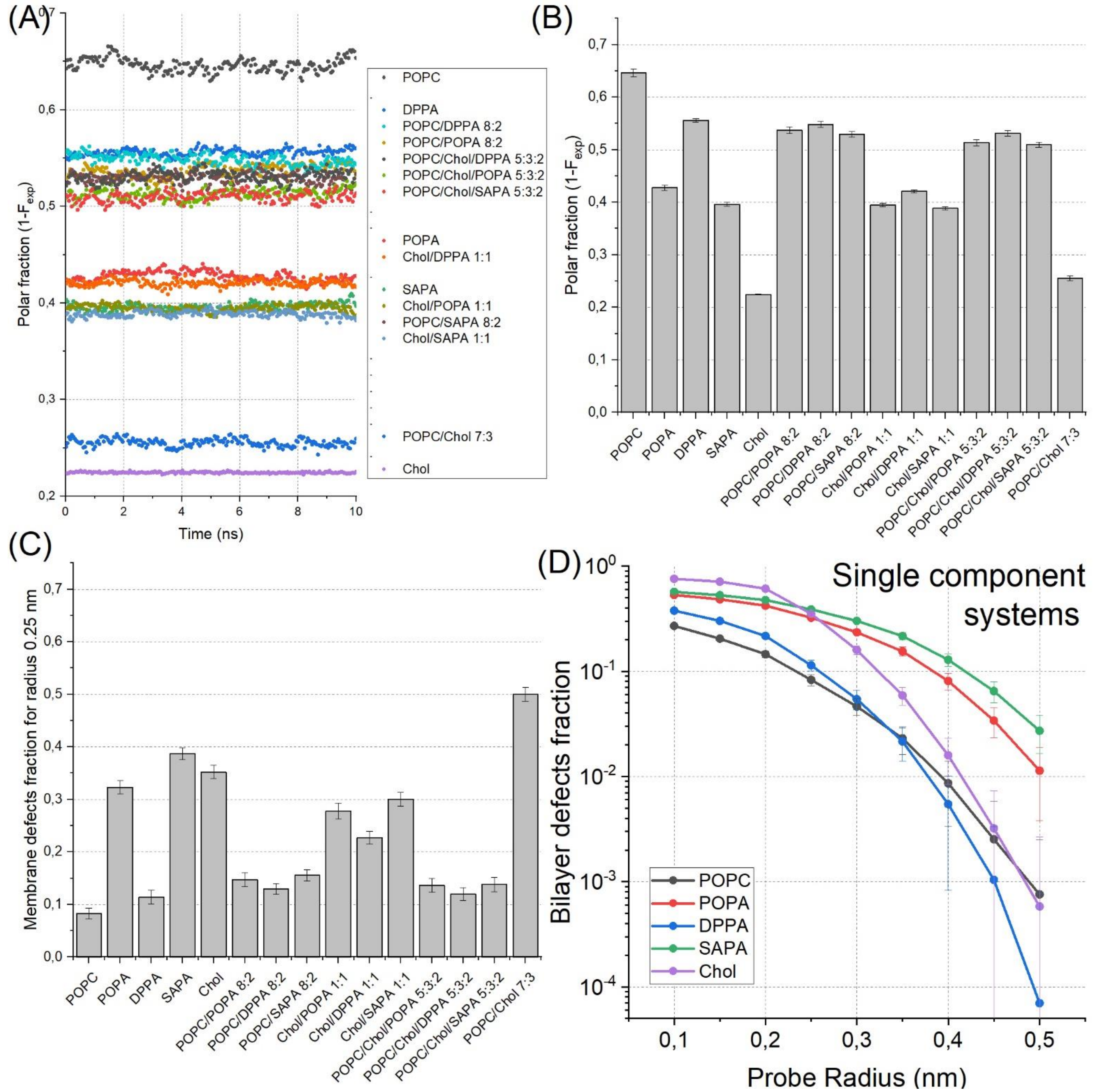

Figure S3. More detailed information regarding membrane defect analysis. In panel (A) evolution in time of polar fraction (1-F_{exp}) is presented for all of investigated systems. In panel (B) average values and standard deviations of polar fraction are calculated and visualized. In panel (C) membrane defects fraction probed with radius 0.25 nm are presented. In panel (D) change in bilayer defects fraction for single component systems is presented.

2. Langmuir Monolayer

2.1 Individual surface Pressure−Area (π−A) Isotherms

Below are presented individual surface pressure-area isotherms of investigated lipid monolayers. Monolayers formed from single lipids are presented in figure S4 panels A-E. Additional isoform of POPC/Chol was presented in figure S4 panel F. Isotherms formed from POPC:PA lipid compositions are presented in figure S5. Results for monolayers formed from Cholesterol:PA compositions are presented in figure S6. Isotherms formed from three lipids are presented in figure S7.

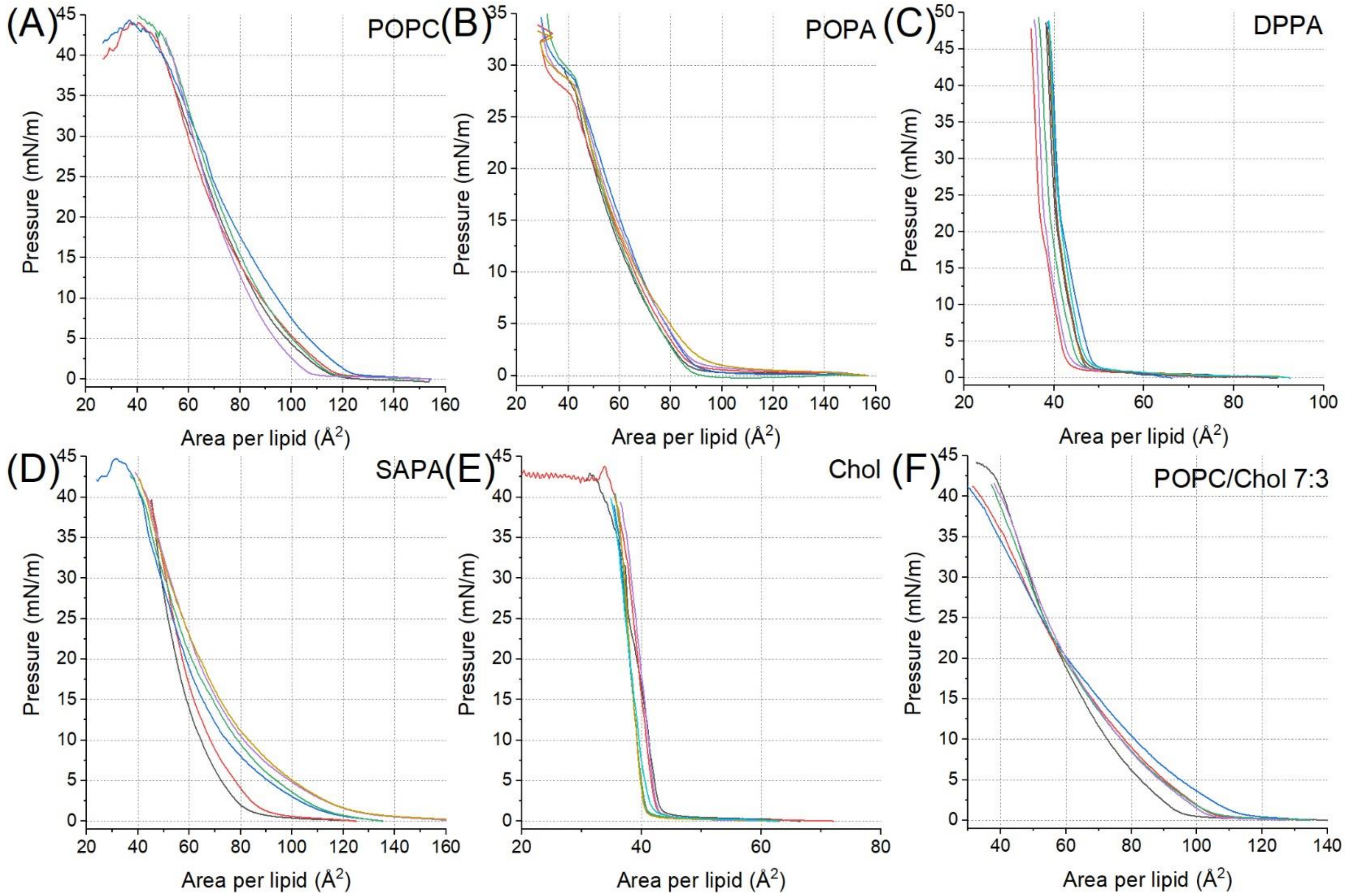

Figure S4. Individual monolayers surface pressure-area compression isotherms of single lipid compositions for (A) POPC, (B) POPA, (C) DPPA, (D) SAPA, (E) Cholesterol single component systems.

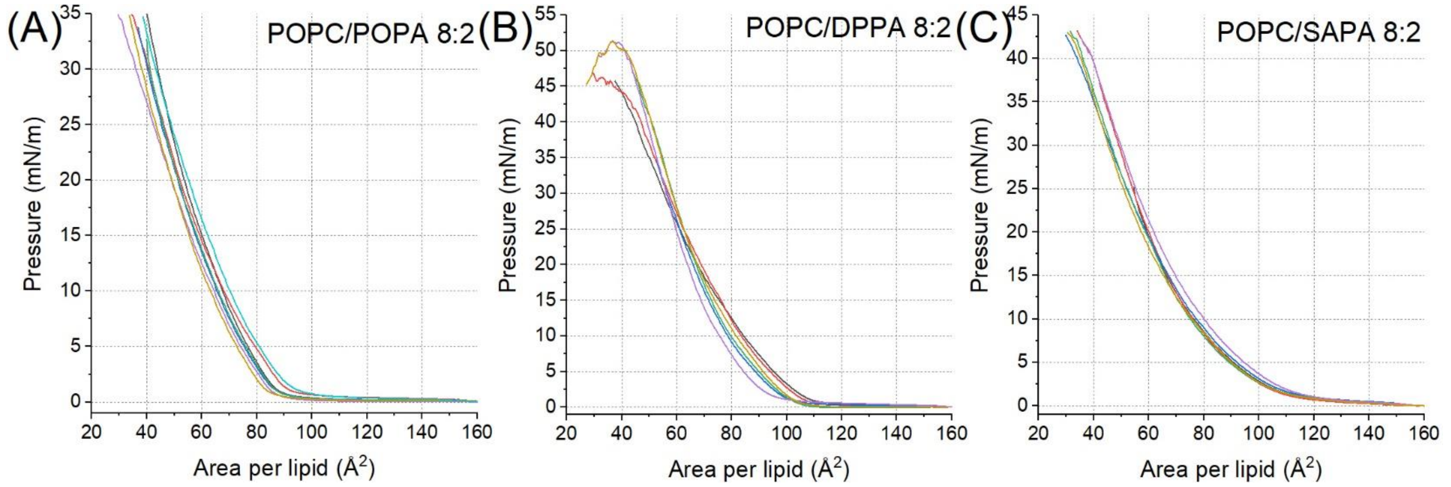

Figure S5. Individual monolayers surface pressure-area compression isotherms for (A) POPC:POPA, (B) POPC:DPPA, (C) POPC:SAPA of POPC:PA (8:2) lipid compositions.

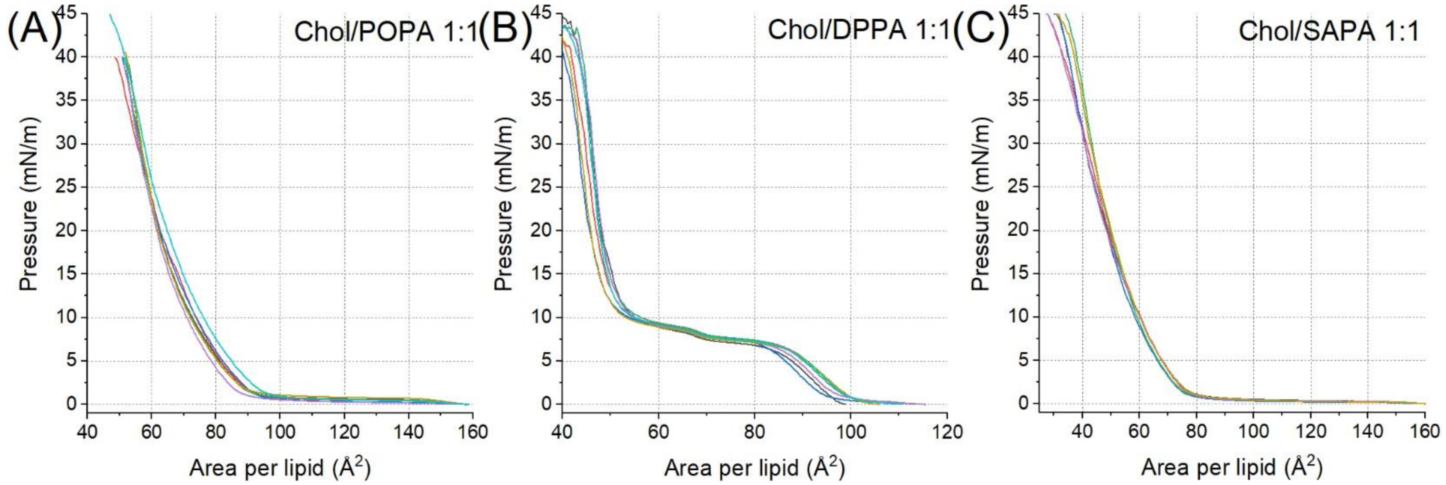

Figure S6. Individual monolayers surface pressure-area compression isotherms for (A) Chol:POPA, (B) Chol:DPPA, (C) Chol:SAPA of Chol:PA (1:1) lipid compositions.

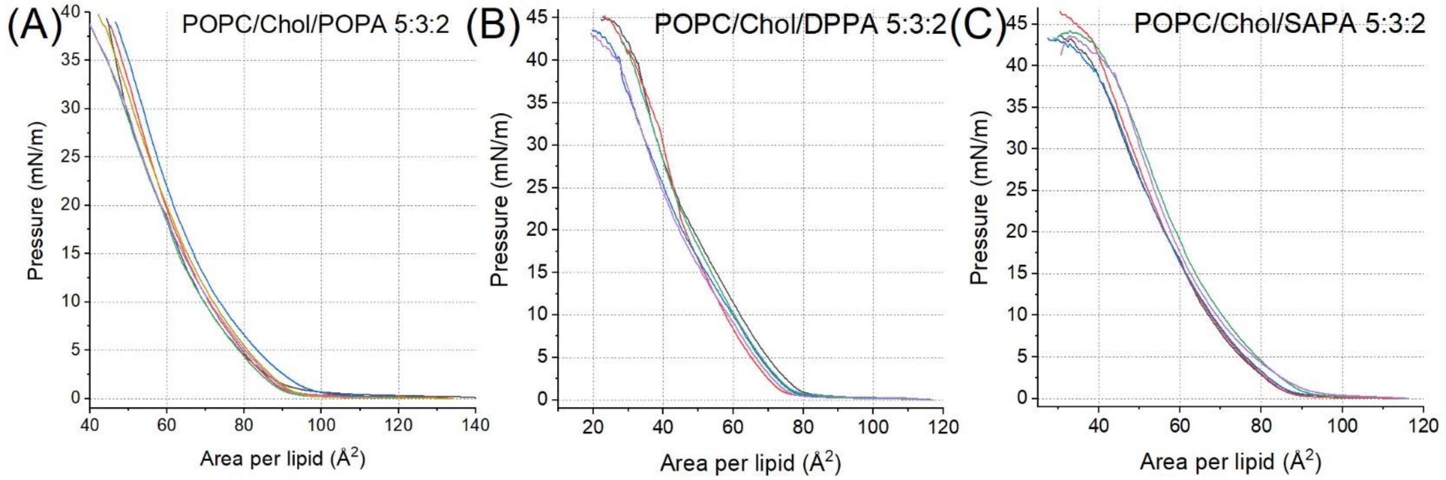

Figure S7. Individual monolayers surface pressure-area compression isotherms for). (A) POPC:Chol:POPA, (B) POPC:Chol:DPPA, (C) POPC:Chol:SAPA of POPC:Cholesterol:PA (5:3:2) lipid compositions.

2.2 Statistical significance of APL

In table S.2 a statistical significance results is presented of APL measurements.

Tab S.2. Q-values for APL comparison using Tukey-test. Results statically significant are marked with green dot, while not significant with red dot.

APL Tukey-test (q Value)	POPC	POPA	DPPA	SAPA	Chol	POPC/POPA 8:2	POPC/DPPA 8:2	POPC/SAPA 8:2	Chol/POPA 1:1	Chol/DPPA 1:1	Chol/SAPA 1:1	POPC/Chol/ POPA 5:3:2	POPC/Chol/ DPPA 5:3:2	POPC/Chol/ SAPA 5:3:2
POPA	🟢 12,3													
DPPA	🟢 29,8	🟢 18,0												
SAPA	🟢 16,1	🔴 4,6	🟢 12,4											
Chol	🟢 29,4	🟢 17,6	🔴 0,4	🟢 12,1										
POPC/POPA 8:2	🟢 29,8	🟢 18,4	🔴 1,0	🟢 12,9	🔴 1,4									
POPC/DPPA 8:2	🟢 6,3	🟢 5,7	🟢 23,0	🟢 9,8	🟢 22,6	🟢 23,2								
POPC/SAPA 8:2	🟢 18,7	🟢 8,0	🟢 7,8	🔴 3,6	🟢 7,4	🟢 8,4	🟢 12,8							
Chol/POPA 1:1	🟢 25,3	🟢 13,3	🔴 4,9	🟢 7,9	🔴 4,5	🟢 5,8	🟢 18,5	🔴 3,6						
Chol/DPPA 1:1	🟢 21,2	🟢 9,0	🟢 9,4	🔴 3,8	🟢 9,0	🟢 10,1	🟢 14,4	🔴 0,3	🔴 4,5					
Chol/SAPA 1:1	🟢 28,0	🟢 16,5	🔴 0,9	🟢 11,2	🔴 0,5	🔴 1,8	🟢 21,4	🟢 6,8	🔴 3,9	🟢 8,2				
POPC/Chol/POPA 5:3:2	🟢 15,5	🔴 3,4	🟢 14,5	🔴 1,3	🟢 14,1	🟢 14,9	🟢 9,0	🔴 4,9	🟢 9,7	🟢 5,4	🟢 13,1			
POPC/Chol/DPPA 5:3:2	🟢 31,7	🟢 20,9	🔴 4,4	🟢 15,6	🔴 4,8	🔴 3,4	🟢 25,4	🟢 11,1	🟢 8,9	🟢 13,0	🟢 5,1	🟢 17,6		
POPC/Chol/SAPA 5:3:2	🟢 17,7	🟢 5,7	🟢 12,1	🔴 0,8	🟢 11,8	🟢 12,7	🟢 11,1	🔴 2,9	🟢 7,4	🔴 3,1	🟢 10,9	🔴 2,2	🟢 15,5	
POPC/Chol	🟢 17,6	🟢 7,7	🟢 6,6	🔴 3,7	🟢 6,3	🟢 7,3	🟢 12,2	🔴 0,4	🔴 2,8	🔴 0,7	🟢 5,8	🔴 4,9	🟢 9,8	🔴 3,1

2.3 Excess free energy of mixing (ΔG_m^{ex})

Below are presented the visualization of mixing behaviour for investigated monolayer systems. Two component systems are presented in figures S7 and S8 panel (A). Visualization of all single-component systems is presented in figure S8 panel (B). Visualization of three-component systems is presented in figure S9.

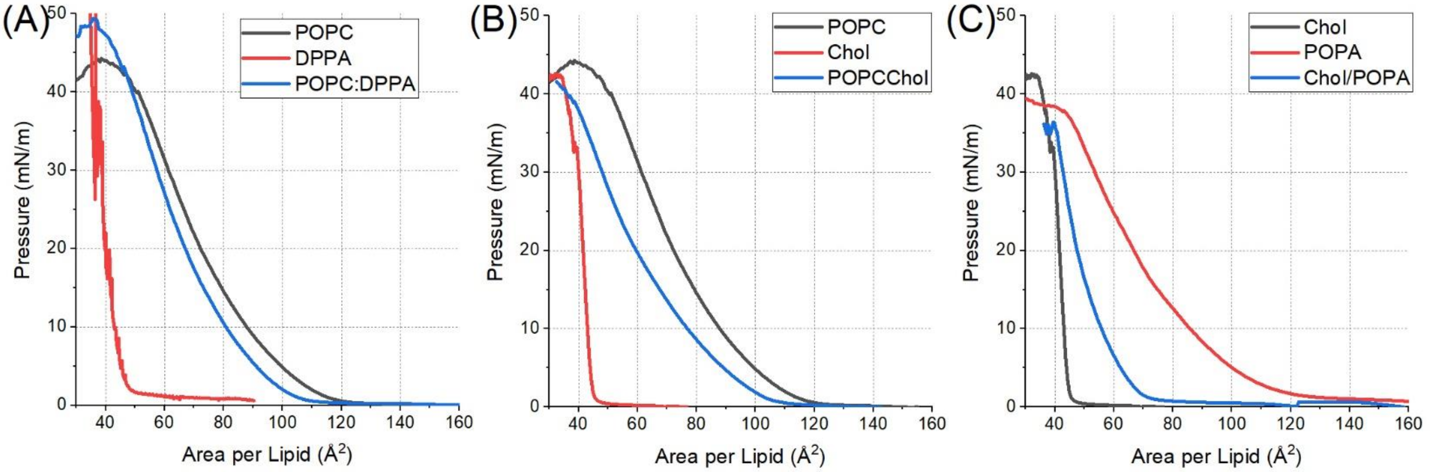

Figure S8. Visualization of monolayers isotherms of (A) POPC, DPPA and POPC/DPPA (8:2) systems, (B) POPC, Chol and POPC/Chol (7:3) systems, (C) Chol, POPA and Chol/POPA (1:1) systems.

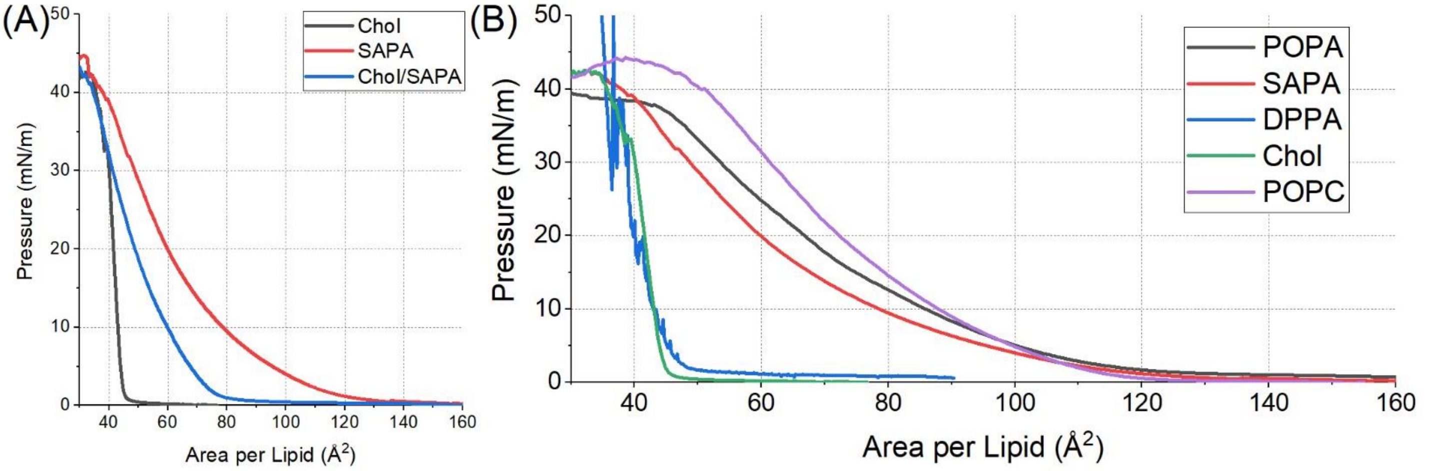

Figure S9. Visualization of monolayers isotherms of (A) POPC, SAPA and Chol/SAPA (1:1) systems, (B) single component monolayers averaged isotherms.

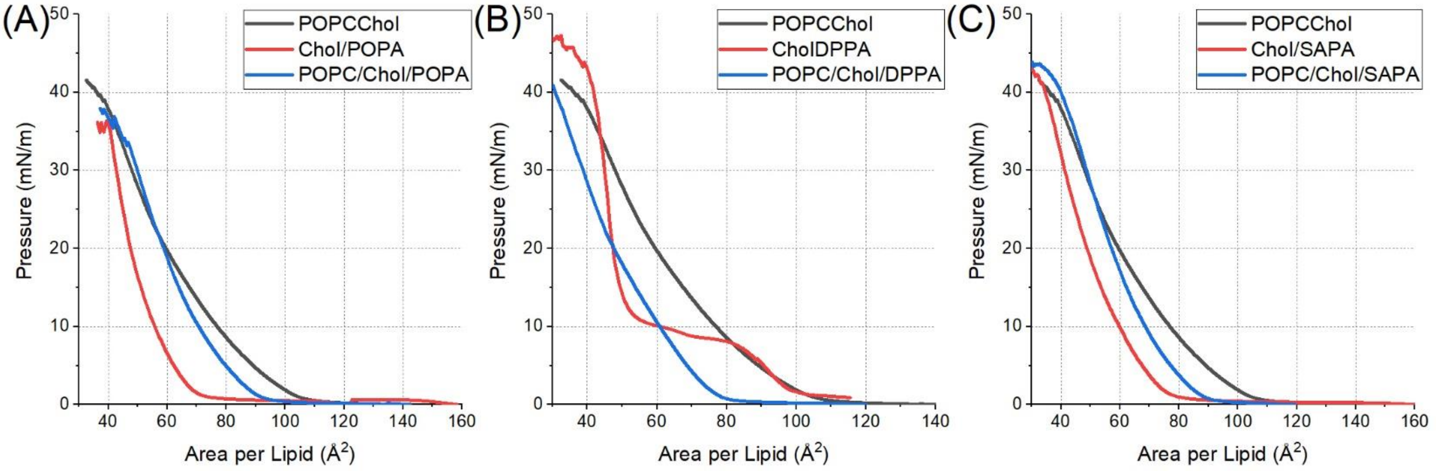

Figure S10. Visualization of monolayers isotherms of (A) POPC/Chol (8:3), Chol/POPA (1:1) and POPC/Chol/POPA (5:3:2) systems, (B) POPC/Chol (8:3), Chol/DPPA (1:1) and POPC/Chol/DPPA (5:3:2) systems, (A) POPC/Chol (8:3), Chol/SAPA (1:1) and POPC/Chol/SAPA (5:3:2) systems.

3. Flicker Noise Spectroscopy

In figures S11-S17 detailed information for determination of bending rigidity coefficient of investigated systems was presented. In each figure a panel A with results and error for individual measurements using both approaches – average-based and statistical – is presented. This is followed by averaged values for both of those methods, respectively. Additionally, an example snapshot of investigated vesicle is presented in panel B. Finally, a

population of bending rigidity based on Kernel based approximation of density function is presented is presented in panel C. Do note two populations of bending rigidity in the case of POPC/DPPA (figure S12), which could indicate inhomogeneous dispersion of lipids in the membrane.

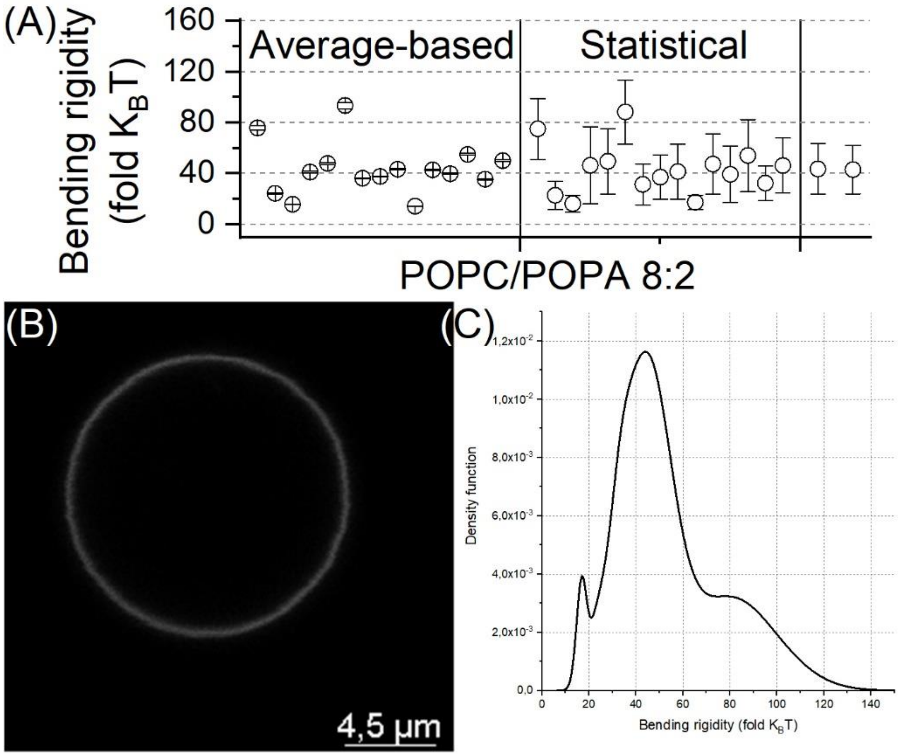

Fig S11. (A) Individual bending rigidity measurements for POPC/POPA with two approaches – averaged-based and statistical. In third quarter of the plot average values are presented. (B) Example of recorded GUV. (C) Kernel density function based on bending rigidity values obtained from statistical approach.

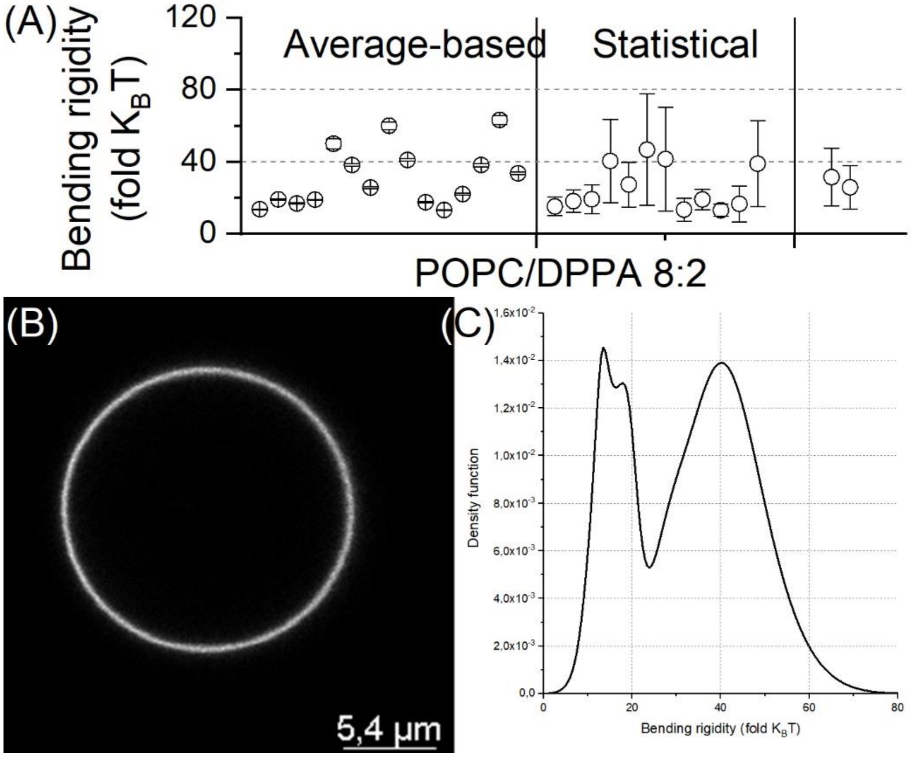

Fig S12. (A) Individual bending rigidity measurements for POPC/DPPA with two approaches – averaged-based and statistical. In third quarter of the plot average values are presented. (B) Example of recorded GUV. (C) Kernel density function based on bending rigidity values obtained from statistical approach.

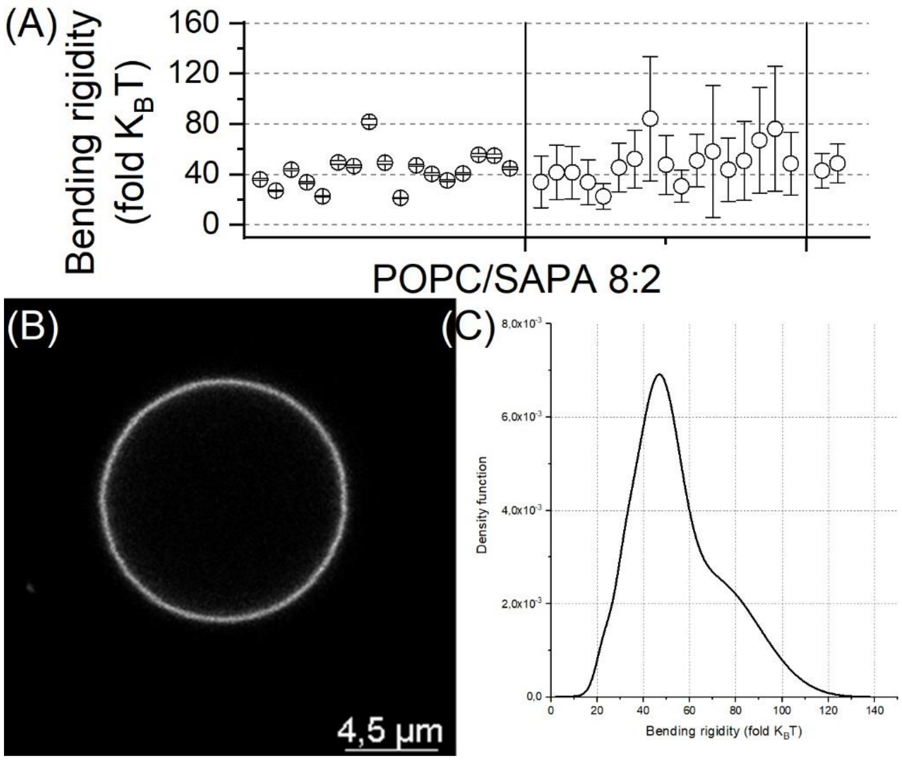

Fig S13. (A) Individual bending rigidity measurements for POPC/SAPA with two approaches – averaged-based and statistical. In third quarter of the plot average values are presented. (B) Example of recorded GUV. (C) Kernel density function based on bending rigidity values obtained from statistical approach.

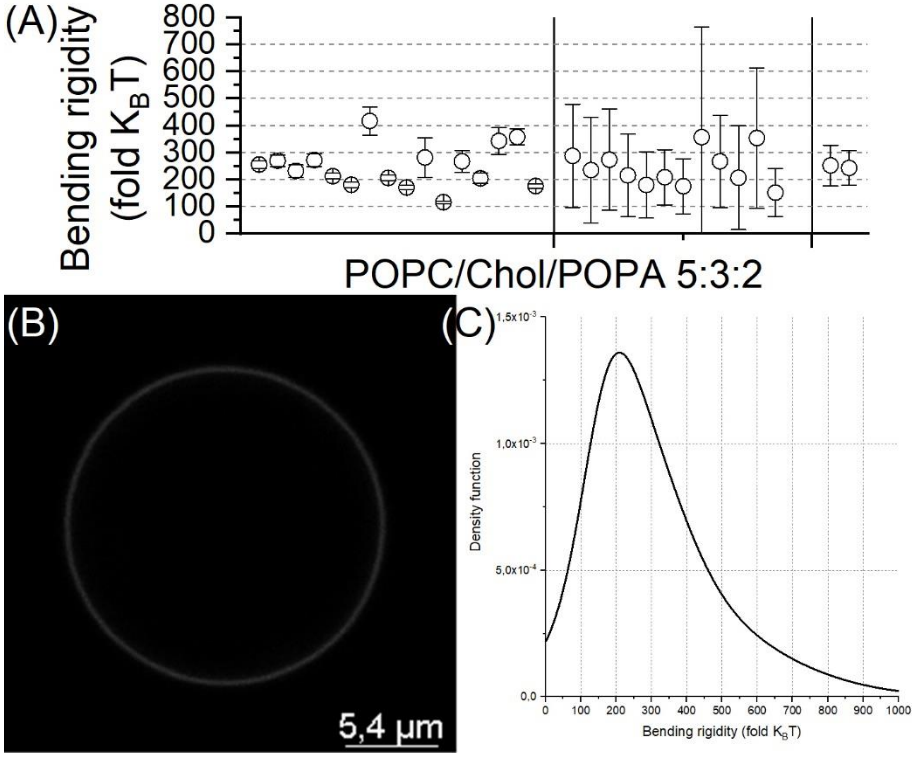

Fig S14. (A) Individual bending rigidity measurements for POPC/Chol/POPA with two approaches – averaged-based and statistical. In third quarter of the plot average values are presented. (B) Example of recorded GUV. (C) Kernel density function based on bending rigidity values obtained from statistical approach.

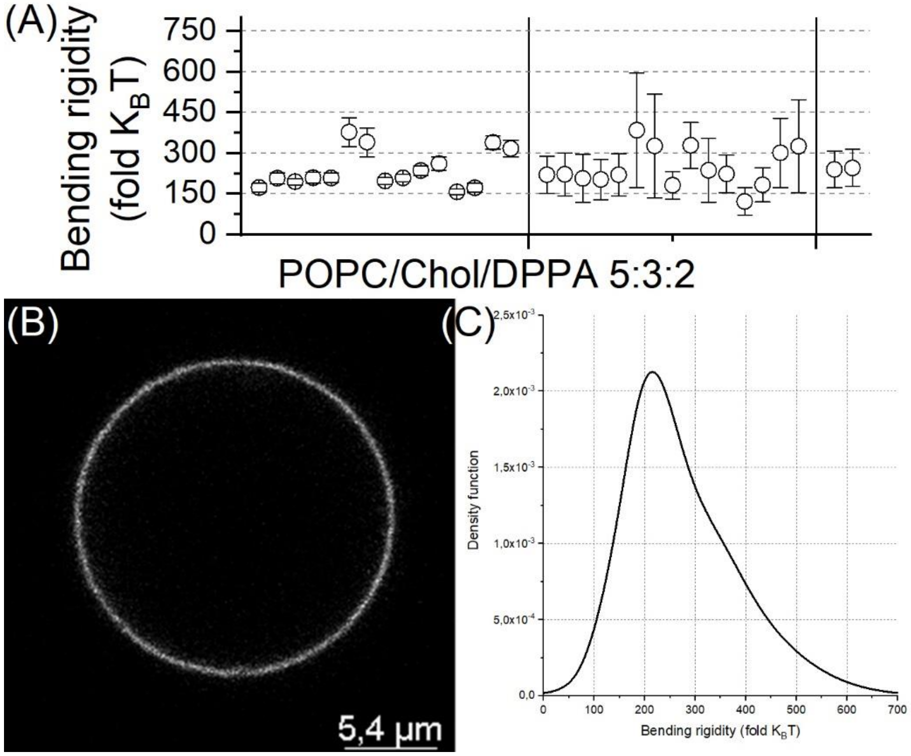

Fig S15. (A) Individual bending rigidity measurements for POPC/Chol/DPPA with two approaches – averaged-based and statistical. In third quarter of the plot average values are presented. (B) Example of recorded GUV. (C) Kernel density function based on bending rigidity values obtained from statistical approach.

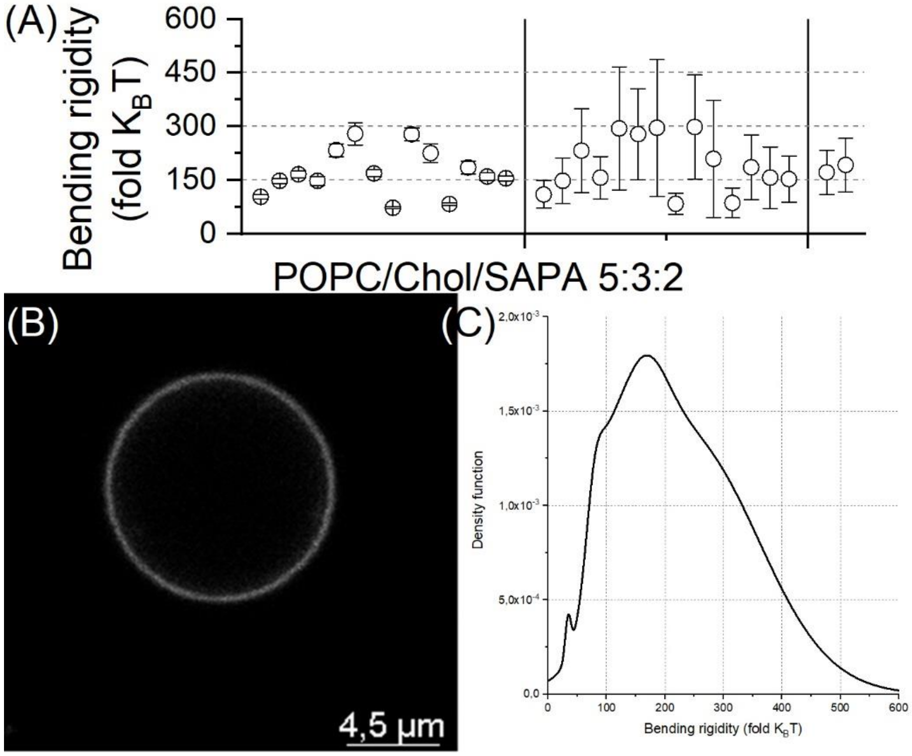

Fig S16. (A) Individual bending rigidity measurements for POPC/Chol/SAPA with two approaches – averaged-based and statistical. In third quarter of the plot average values are presented. (B) Example of recorded GUV. (C) Kernel density function based on bending rigidity values obtained from statistical approach.

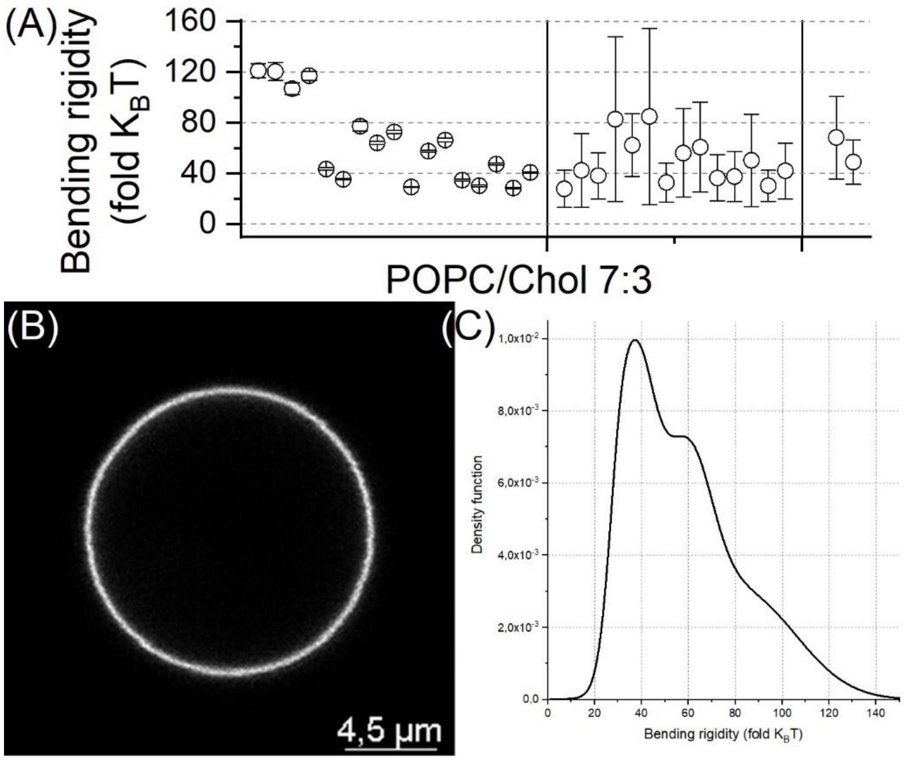

Fig S17. (A) Individual bending rigidity measurements for POPC/Chol with two approaches – averaged-based and statistical. In third quarter of the plot average values are presented. (B) Example of recorded GUV. (C) Kernel density function based on bending rigidity values obtained from statistical approach.
